# Eco-Friendly and High-Performance Bio-Polyurethane Adhesives from Vegetable Oils: A Review

**DOI:** 10.3390/polym16111613

**Published:** 2024-06-06

**Authors:** Sena Maulana, Eko Setio Wibowo, Efri Mardawati, Apri Heri Iswanto, Antonios Papadopoulos, Muhammad Adly Rahandi Lubis

**Affiliations:** 1 Department of Forestry Engineering, Institut Teknologi Sumatera (ITERA), Bandar Lampung 35365, Indonesia; sena001@brin.go.id; 2 Research Center for Biomass and Bioproducts, National Research and Innovation Agency, Bogor 16911, Indonesia; ekos009@brin.go.id; 3 Department of Agro-Industrial Technology, Universitas Padjadjaran, Bandung 40600, Indonesia; efri.mardawati@unpad.ac.id; 4 Research Collaboration Center for Biomass and Biorefinery between BRIN and Universitas Padjadjaran, Bandung 40600, Indonesia; 5 Department of Forest Products Technology, Faculty of Forestry, Universitas Sumatera Utara, Medan 20355, Indonesia; apri@usu.ac.id; 6 Laboratory of Wood Chemistry and Technology, Department of Forestry and Natural Environment, Democritus University of Thrace, GR-66100 Drama, Greece

**Keywords:** bio-based adhesives, eco-friendly, sustainable, polyurethane, vegetable oils

## Abstract

Current petrochemical-based adhesives adversely affect the environment through substantial volatile organic compound (VOC) emissions during production, contributing to air pollution and climate change. In contrast, vegetable oils extracted from bio-resources provide a compelling alternative owing to their renewability, abundance, and compatibility with adhesive formulation chemistry. This review aimed to critically examine and synthesize the existing scholarly literature on environmentally friendly, sustainable, and high-performance polyurethane adhesives (PUAs) developed from vegetable oils. The use of PUAs derived from vegetable oils promises to provide a long-term replacement while simultaneously maintaining or improving adhesive properties. This quality renders these adhesives appropriate for widespread use in various sectors, including construction, automotive manufacturing, packaging, textile, and footwear industries. This review intended to perform a comprehensive assessment and integration of the existing research, thereby identifying the raw materials, strengths, weaknesses, and gaps in knowledge concerning vegetable oil-based PUAs. In doing so, it responded to these gaps and proposes potential avenues for future research. Therefore, this review accomplishes more than merely evaluating the existing research; it fosters the advancement of greener PUA technologies by identifying areas for improvement and innovation towards more sustainable industrial practices by showcasing vegetable oil-based PUAs as viable, high-performance alternatives to their petroleum-based counterparts.

## 1. Introduction

Recently, there has been a growing worldwide recognition and interest regarding the ecological effects of traditional adhesives. These adhesives, commonly derived from petrochemical sources, result in air contamination and climate fluctuation as they release volatile organic compounds (VOCs). In the US, VOC emissions totaled 12,351 tons in 2023 throughout all production processes, such as the application and curing stages [1,2,3]. The presence of VOCs not only spoils air quality but also causes negative impacts on human health, such as respiratory irritation, sensory irritation, and sick-building syndrome, and in the long-term, it can be carcinogenic [4,5,6,7]. Consequently, addressing these environmental problems through the advancement of greener substitutes is more critical now than ever [8]. As a response to this issue, scientists and industries have initiated novel approaches toward creating eco-friendly adhesives that are both sustainable and efficient simultaneously [9,10].

An optimistic approach to addressing the environmental impact of polyurethane adhesives (PUAs) is to employ vegetable oils as feedstock for their production. Vegetable oils from diverse sources like soybeans [11,12,13,14,15,16,17], the castor plant [16,18,19,20,21,22,23,24,25,26], and linseeds [27,28] bring several benefits, such as being abundant, renewable, and featuring appropriate chemical properties for adhesive formulations [18,19,21,26,29,30,31,32,33,34,35,36,37,38,39,40,41,42]. Substituting petrochemical-based materials with vegetable oils could reduce dependence on fossil fuels while minimizing the carbon footprint associated with using and producing these adhesives [43].

The importance of using eco-friendly PUAs made from vegetable oils can be attributed to their capacity to offer a sustainable substitute while retaining or enhancing adhesive properties [44,45]. These types of adhesives possess advantageous features, such as exceptional bonding strength, malleability, and longevity, that make them appropriate for use in various sectors.

The need for environmentally friendly adhesives has surged due to both ecological and sustainable practices in many industries. Examples of such sectors include construction [46,47,48,49,50], automotive [51], packaging [52], textiles [53,54], footwear [9,55], which are pursuing alternative options that meet their sustainability objectives and regulatory stipulations. To minimize environmental effects while advancing economic ambitions in these fields, the utilization of eco-friendly adhesives has become a viable solution [56].

To fully exploit their potential, it is important to thoroughly understand the current status of research on eco-friendly, sustainable, and high-performing PUAs derived from vegetable oils. This literature review aimed to critically analyze and synthesize existing scientific publications to identify deficiencies, strengths, and knowledge gaps. By filling these voids and presenting suggestions for future directions along with challenges faced in this field, this critique will advance progress toward ecological adhesive technologies becoming more prevalent in adoption.

This review aimed to thoroughly analyze current research on environmentally friendly, sustainable, and high-performing PUAs derived from vegetable oils by critically evaluating existing scientific publications available in global databases, including research articles, conference papers, and other relevant sources. Various aspects of these adhesives were explored, such as the formulation techniques used for their development, along with the raw materials and additives employed in the process that affect adhesive properties. Apart from performance characteristics like strength and durability, emphasis was also on resistance against environmental factors.

## 2. Vegetable Oil-Based Adhesives: An Overview

### 2.1. Definition and Classes of Adhesives

In material bonding, adhesives are fundamental to creating cohesive and adhesive joints capable of withstanding rigorous demands. They offer diverse advantages such as versatility, user-friendliness, and inter-material binding capabilities spanning metal composites to plastics [13,18,54,57,58]. Adhesives can be categorized into several classes as per the classification by Pizzi and Mittal [59]. Some of these categories include protein adhesives, rubber-based adhesives, elastic adhesives, phenolic resin adhesives, resorcinol and phenol-resorcinol adhesives, natural phenolic adhesives derived from tannins and lignin, urea and melamine amino resin adhesives, PUAs, reactive acrylic adhesives, anaerobic adhesives, aerobic acrylic adhesives, bio-based acrylic adhesives, silicone adhesives and sealants, epoxy adhesives, bio-sourced epoxy monomers and polymers, and pressure-sensitive adhesives. Table 1 offers a comparative overview of these adhesive classes, focusing on their key components, primary advantages and drawbacks, and significant applications. Each adhesive class has unique properties that serve specific application requirements, illustrating the versatility of adhesives.

The market is dominated by a wide variety of adhesives, many of which are synthetic and fossil-fuel-based. However, the dwindling fossil fuel resources and the focus on sustainable materials have led to increased interest in an eco-friendly alternative: vegetable oil-based adhesives [9,18,19,21,26,27,29,30,32,33,34,35,36,37,39,40,41,42,73,74], lignin-based adhesives [75,76], tannin-based adhesives [76,77], protein-based adhesives [78,79], and starch-based adhesives [80,81,82].

Lignin and tannin from various renewable materials are currently also being developed as raw materials for making PU [75,76]. However, lignin-based PUs exhibit higher brittleness due to the rigid and complex aromatic structure of lignin, which limits the flexibility and ductility of the resulting polymer matrix [75]. Vegetable oil-based PUs can potentially overcome brittleness due to their aliphatic structure, which provides greater flexibility and elasticity compared to the aromatic structure of lignin, resulting in a more ductile and less brittle polymer matrix [83,84]. Lignin and tannin-based PUs often suffer from poor water resistance due to the hydrophilic nature of lignin’s hydroxyl groups, which can limit their application in environments with high moisture exposure [76]. Vegetable oil-based PUs, on the other hand, generally exhibit better hydrophobicity and water resistance compared to lignin, making them more suitable for applications requiring moisture barrier properties [84,85].

These vegetable oil-based adhesives incorporate renewable sources like soybean oil [11,12,13,14,15,16,17,86,87], castor oil [16,18,19,20,21,22,23,24,25,26], linseeds [27,28], and others into their manufacturing processes. The triglycerides in these oils undergo chemical modifications to create PU-based adhesives that have suitable qualities for diverse applications [10]. Vegetable-oil-based adhesives display impressive properties, making them appealing for various industrial applications. These properties encompass reliable bonding ability on a range of substrates, such as wood or metal. They also show enhanced biodegradability compared to traditional adhesives originating from fossil fuels [47]. Using plant-sourced materials reduces reliance on non-renewable resources, supporting sustainability goals through circular economy principles [9].

### 2.2. Advantages and Challenges of Vegetable Oil-Based Adhesives

Recent literature discusses the benefits of using vegetable oil-based adhesives as an eco-friendly alternative to traditional formulations. In particular, their renewable and biodegradable nature, low toxicity, versatility, improved properties, and scope for customization make them a viable option in adhesive manufacturing. A thorough evaluation of these advantages and challenges is provided in this section.

#### 2.2.1. Advantages of Vegetable Oil-Based Adhesives

Vegetable oil-based adhesives yield remarkable advantages, predominantly linked to their renewable origin. These adhesives are derived from plant-based oils such as soybean oil [11,12,13,14,15,16,17,86,87], castor oil [16,18,19,20,22,23,24,25,26], linseed [27,28], and others. This distinguishes them from petroleum-based adhesives, which rely on non-renewable fossil fuel resources that face depletion. In contrast, the vegetable oils used to produce these adhesives can be continually replenished through sustainable agricultural practices, thus positioning them as an environmentally sustainable solution.

Arguably, vegetable oil-based adhesives’ most significant advantage is their ecological footprint. Their production reduces dependence on non-renewable resources and contributes to an eco-friendly manufacturing process [10,88]. Additionally, these adhesives possess the desirable qualities of biodegradability, low toxicity, and versatility, making them an attractive option for various applications across industries. They also offer the potential for customization, presenting tremendous opportunities for innovations in adhesive technology. These merits collectively render vegetable oil-based adhesives a viable and sustainable alternative to conventional adhesive formulations.

Numerous studies have shown that vegetable oil-based adhesives exhibit comparable, and in some instances, superior, performance and characteristics to conventional petroleum-based analogs. They possess robust adhesion properties, exquisite flexibility, and excellent compatibility with various substrates [19,21,29,31,32,34,40,54,89,90,91]. Their inherent elasticity makes them suitable for applications demanding high elongation and high-strength bonding, such as in construction [46,47,48,49,50], automotive [51], packaging [52], textiles [53,54], footwear [9,55], and electronics [29] industries. The mechanical properties of these adhesives can be tailored by modifying experimental parameters, the choice of vegetable oil, or the incorporation of additives, thus allowing for the design of application-specific adhesives [27,86,92]. For instance, Zhang et al. [27] reported that through the judicious selection of both the oil source and curing temperature, the resulting adhesive’s toughness could be enhanced, yielding a material with high resistance to thermal and mechanical stresses.

Furthermore, the use of vegetable oil-based adhesives has a favorable economic impact. Vegetable oils are traditionally less expensive than their petroleum counterparts [93]. Moreover, the utilization of agricultural waste, such as vegetable oils, also drives local economies and supports rural development by offering potential income avenues for farmers [59,94]. There are noteworthy health and safety benefits, too. Vegetable oil-based adhesives exhibit reduced volatility and lower hazardous emissions compared to traditional adhesives, resulting in safer working environments [95,96]. Their non-toxic nature and potential for recyclability further their appeal from a health, safety, and environmental standpoint.

In conclusion, the advantages of vegetable oil-based adhesives extend beyond environmental sustainability into arenas of superior performance, economic viability, health and safety, and regional development. Their holistic benefits uniquely position them as a viable and attractive sustainable alternative to adhesive technology. With ongoing research resulting in innovative improvements in their formulation and application, vegetable oil-based adhesives indeed hold promise for a more sustainable world.

#### 2.2.2. Environmental Benefits of Vegetable Oil-Based Adhesives

Petroleum-derived adhesives currently hold a significant market share. However, due to the harmful nature of formaldehyde, there has been a push to create alternative adhesives such as those derived from vegetable oil [97]. The adhesives have been extensively researched and found to have significant environmental advantages, including decreased emissions and ecological effects [18,31,90,98,99]. Vegetable oils consist of harmless, easily decomposable, and environmentally friendly components [100,101], resulting in decreased carbon dioxide emissions and diminished VOCs in comparison to traditional adhesives [97,102,103,104]. The emission of VOCs from conventional adhesives has a detrimental impact on air quality and can lead to health problems [105,106].

Adhesives made from vegetable oil reduce greenhouse gas emissions and energy usage during production since they require lower processing temperatures and shorter curing times [32,56,107]. These adhesives undergo spontaneous biodegradation, resulting in the breakdown of their components into harmless substances over a period of months to years. The rate of degradation depends on the specific formulation of the adhesive and the prevailing conditions [36,56]. However, there are still obstacles to overcome in order to enhance the effectiveness and longevity of adhesives made from vegetable oil [108]. Adopting sustainable sourcing and manufacturing processes is crucial in order to reduce the negative effects on the environment, specifically addressing issues such as deforestation and pesticide utilization [97]. Conducting comprehensive lifecycle assessments (LCAs) is crucial in evaluating the overall sustainability of vegetable oil-based adhesives compared to traditional options [109,110]. The research by McDevvit and Grigsby [111] has demonstrated that adhesives derived from bio-based components, such as vegetable oils, had a considerably reduced environmental impact throughout their whole lifespan as compared to adhesives derived from petrochemical sources. For instance, a bioadhesive consisting exclusively of bio-based materials has a 22% lower life cycle effect than a petrochemical adhesive.

#### 2.2.3. Challenges of Vegetable Oil-Based Adhesives

Despite the eco-friendly benefits of adhesives made from vegetable oils, their manufacturing and handling approaches may result in higher expenses compared to synthetic options. These additional costs can come from obtaining and refining the vegetable oils used and the specialized equipment required for processing [30,31,33,41,98,112]. This increased cost could stymie the widespread acceptance of these adhesives, particularly in industries that place affordability above all else.

The performance of vegetable oil-derived adhesives differs from that of synthetic alternatives, which may pose particular challenges. These include lower strength for bonding, decreased longevity, and a lack of resistance to specific environmental factors [32,40,113,114]. Factors like temperature, moisture levels, and surface compatibility can impact how well these adhesives work in various applications [115,116]. Therefore, additional development and optimization are necessary to ensure fit-for-purpose proficiency.

In summary, using vegetable oil-based adhesives presents numerous benefits such as renewability, biodegradability, and reduced toxicity, making them an appealing environmentally friendly option in the adhesive industry. Nonetheless, notable impediments to their extensive implementation include heightened expenses and performance concerns that require attention. Ongoing exploration is crucial to refine their efficacy, minimize production expenditures, and boost their competitive edge across diverse industries.

#### 2.2.4. Application Areas

Conventional adhesives have been widely employed in various industries and applications, such as construction, automotive manufacturing, electronics, and aerospace [29,51,117,118]. Their effective performance has been extensively documented, particularly in areas with stringent requirements. While vegetable oil-based adhesives are gradually gaining prominence as an alternative to conventional types for specific application areas, they still have limited practical use [57,119,120]. They exhibit great potential in sustainability-oriented sectors like green building construction, eco-packaging, and environmentally friendly consumer products. However, they may not be adopted yet where specific performance standards must be met due to existing adherence to well-established conventional adhesive technologies demanding regulatory compliance certifications.

## 3. High-Performance Vegetable Oil-Based PUAs: Preparation and Properties

### 3.1. Raw Materials of Vegetable Oil-Based PUAs

The preparation and properties of high-performance vegetable oil-based PUAs rest heavily on the selection and processing of raw materials. The primary constituents include vegetable oils which, upon conversion into polyols, react with diisocyanates to form PU. Alternatively, for non-isocyanate-based PUAs, amines serve as raw materials [27,87,121,122,123]. Research focusing on renewable alternatives to petroleum-based ingredients has explored a broad range of vegetable oils. These comprise canola oil [31,124,125], corn oil [126], rubber seed oil [30], crude algal oil [127], sunflower oil [124], camelia oil [124], jatropha oil [128,129], and many more. An essential process involves turning the triglycerides in these oils into polyols, which form vital building blocks for PUA creation [130,131].

**Figure 1:**
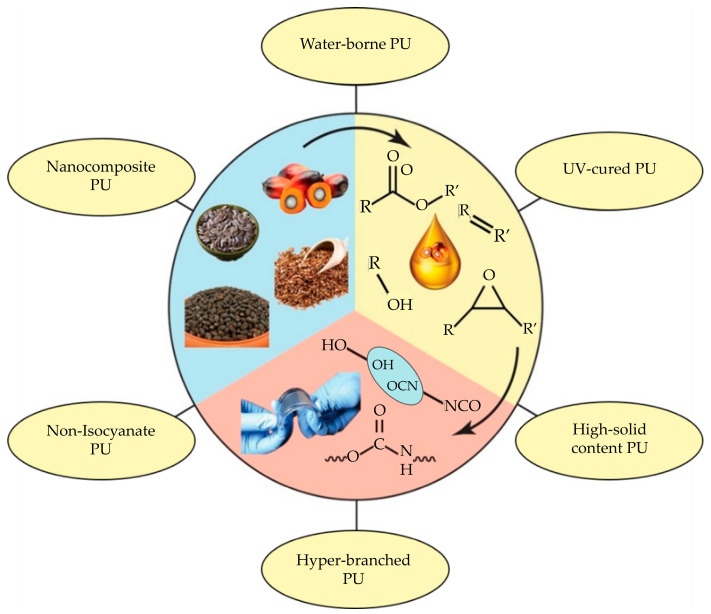
Vegetable oil-based PUA [132]. Adapted with permission from Elsevier, License No. 56185210163.10.

For an effective PUA, the choice of raw materials and their subsequent extraction processes play a pivotal role. Many vegetable oils and extraction techniques have been investigated for polyol synthesis, with processes such as epoxidation, hydroformylation, thiol-ene reaction, ozonolysis, and transesterification being utilized. The extensive research and advancements in this field aim to capitalize on the inherent advantageous properties of vegetable oils. In doing so, they contribute to the development of novel, highly efficient adhesive compositions. The adhesive industry has already embraced environmentally friendly technologies, including ultraviolet (UV)-curable, low- or solvent-free, water-borne, hyperbranched, and high solids content PU adhesives (Figure 1). These advancements are further enhanced by the integration of renewable feedstocks in the synthesis of monomers.

#### 3.1.1. Polyol Extraction Method

Researchers have focused on extracting polyols from vegetable oils in the quest for more sustainable and environmentally friendly approaches in polymer science. This burgeoning field significantly differs from traditional methods, which typically rely on petrochemical raw materials. Various extraction techniques have been explored, each with unique advantages and challenges (Table 2).

**Figure 2:**
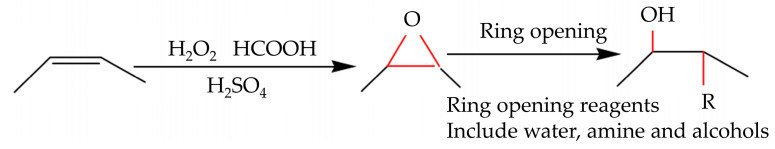
Epoxidation method followed by ring opening for the synthesis of vegetable oil-based polyols [52]. Open access CC BY-NC-ND 4.0.

The epoxidation and oxirane ring-opening method is a critical procedure for turning carbon–carbon double bonds in vegetable oils into epoxy groups, which are vital for polyol synthesis utilized in PU manufacturing (Figure 2). While this process demonstrates the sustainability benefits of employing renewable resources like palm oil, it relies on chemicals such as hydrogen peroxide and formic acid, posing possible environmental dangers if not effectively controlled. Additionally, the methanol used in the process can influence air quality. Although studies like those of Mekewi et al. [52] and Khoon et al. [98] have shown promising results in polyol synthesis using these methods, challenges remain in reducing the environmental footprint due to chemical usage and addressing sustainability concerns associated with palm oil production, including deforestation and environmental damage. Therefore, while the technology offers sustainable options, further research is needed to mitigate its limits and boost its environmental friendliness.

**Figure 3:**
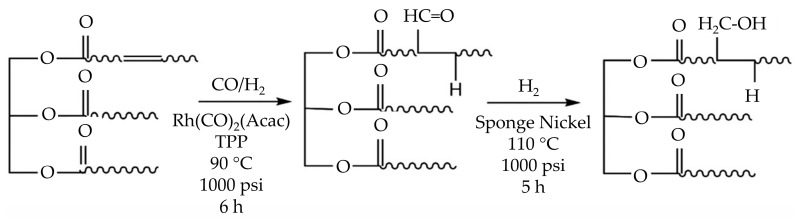
Schematic representation of the polyol manufacturing process by oil hydroformylation [30]. Reprinted/adapted with permission from [30]. John Wiley and Sons, License Number 5615210029478 2019.

In the hydroformylation procedure for generating polyol from rubber seed oil (RSO), Hong et al. [30] utilized a rhodium-based catalyst together with triphenylphosphine (TPP) ligands to create aldehyde molecules with desirable carbonyl (C=O) and hydroxyl (OH) groups, as illustrated in Figure 3. This approach resulted in a polyol with a high hydroxyl number (240 mg KOH g^−1^), indicating good qualities. However, the polyol exhibited a light brown tint, suggesting the necessity for extra processing steps for specific applications. While the research shows the promise of RSO as a renewable raw material, it also raises issues over the environmental impact of employing rhodium and nickel catalysts, considering their rarity, high cost, and risk of water contamination. Future studies could examine more ecologically friendly catalyst choices to boost sustainability.

In Petrović et al.’s [127] study, hydroformylation was employed to transform crude algal oil into polyols, a significant improvement in sustainable polymer material supply. Despite attaining a high OHV of 147 mg KOH g^−1^, indicating it is suitable for foam applications, the resultant polyol had a dark color due to remaining impurities. The study’s use of solvents like toluene and isopropanol poses environmental risks if not managed properly, necessitating a full investigation of the process’s environmental effects. Future efforts should focus on enhancing technologies for sustainable production and maintaining transparency regarding environmental implications throughout the process.

**Figure 4:**
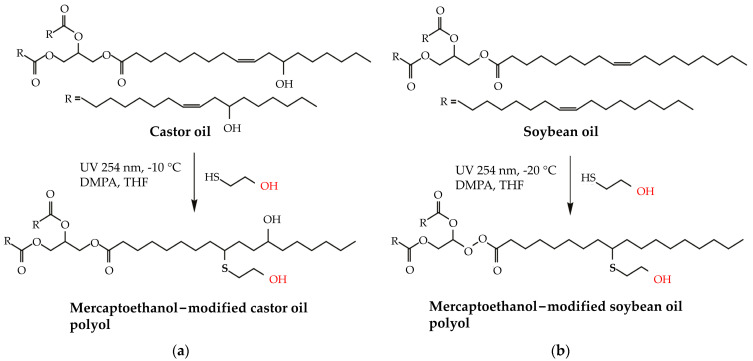
Schematic preparation of castor oil-based polyols (**a**) soybean oil (**b**) by photo-induct thiol-ene reaction method. The red color presents the hydroxyl (OH) group in castor oil-based polyols [16]. Reprinted/adapted with permission from [16]. Elsevier, License Number 5615271216915 2016.

Innovative research by Alagi et al. [16] and Feng et al. [13] addresses the utilization of thiol-ene reactions to manufacture polyols from renewable biomass like castor and soybean oils. The experimental approach depended on a photoinduced thiol-ene reaction and was accomplished using castor oil and soybean oil variations, as indicated in Figure 4. Alagi et al. reported high OHVs (278 mg KOH g^−1^ for castor oil and 203 mg KOH g^−1^ for soybean oil), indicating possible applications in adhesives. However, the low reaction temperatures (−10 °C for castor oil and −20 °C for soybean oil) and extended reaction durations create scalability and cost difficulties. Similarly, Feng et al. utilized soybean oil with eco-friendly methods; however, the use of mercury lamps poses environmental problems. While these approaches offer sustainable options, further study is needed to optimize reaction conditions, eliminate environmental concerns, and enhance overall efficiency.

Another significant study by Ionescu et al. [134] suggested greener options using thiol-ene reactions to create polyols from castor oil. They produced high OHVs (286 mg KOH g^−1^ for castor oil-ME and 258 mg KOH g^−1^ for MCO-AA) with acceptable viscosities and low hazardous waste. However, the use of potentially harmful photo-initiators shows room for improvement in environmental effects. Future research should focus on safer alternatives and more efficient extraction procedures to reach the full promise of these eco-friendly methodologies in polyol synthesis.

**Figure 5:**
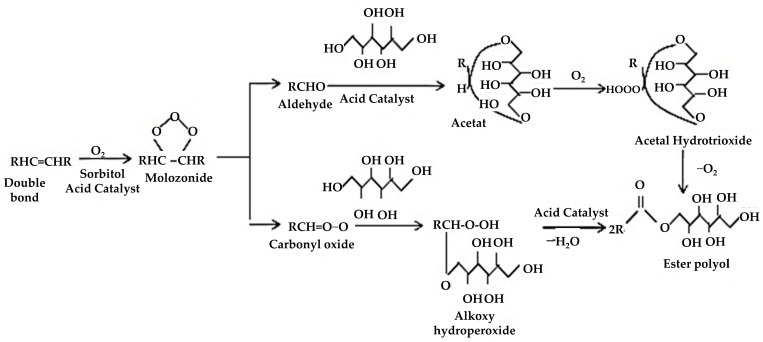
Ozonolysis mechanism for polyol synthesis [94]. Open access CC BY-NC-ND 4.0.

Ozonolysis is a process for manufacturing polyols from seed oil, involving the interaction of fatty acid carbon double bonds with ozone to generate ozonide rings, which are subsequently degraded into aldehyde and hydroxyl groups. The mechanism of polyol production via the ozonolysis method is given in Figure 5. In PU manufacturing, this procedure generates main OH groups with increased mechanical characteristics and higher glass transition temperatures. Dumont et al. [124] carried out research on polyol synthesis utilizing ozonolysis and hydrogenation, attaining high OH levels in mustard polyols but with potential problems surrounding acid generation during ozonolysis, which could interfere with PU creation. The study emphasized the use of crude vegetable oil for environmental benefits, but it lacked an in-depth evaluation of these improvements. Addressing acid formation optimization and providing a full environmental assessment is critical to the industrial feasibility of this technique. Similarly, another study performed ozonolysis on leftover cooking oil, offering green feedstock advantages but needing careful consideration of safety standards, potential pollutants, and overall process capability for scaling up. Future studies should prioritize cost reduction, environmental effect minimization, and compliance with waste management standards for industrial deployment.

**Figure 6:**
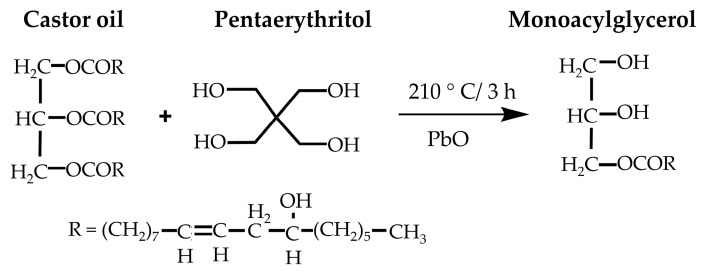
Transesterification reaction of castor oil with pentaerythritol [22]. Open access CC BY-NC-ND 4.0.

Transesterification, a key method for synthesizing polyols from castor oil, involves replacing ester groups with hydroxyl groups from pentaerythritol (Figure 6). Valero and Gonzalez [23] and Das et al. [22] conducted studies using this method, showcasing increased OHV in polyols compared to castor oil. However, optimization of reaction conditions, purity assessment of castor oil, and potential allergenicity and toxicity of pentaerythritol require further exploration. Agrawal et al. [133] also utilized transesterification, achieving high OHV polyols for foam production. The environmental impact of catalysts and chemicals used in transesterification processes warrants scrutiny for energy consumption and emissions. Assessing greener chemical and process alternatives is critical for sustainable polyol production.

A thorough evaluation of polyol extraction methods from vegetable oils indicates exciting developments as well as substantial obstacles in the realm of green chemistry. Epoxidation and oxirane ring opening technologies indicate that utilizing palm oil has sustainability benefits, but they rely on potentially dangerous substances such as hydrogen peroxide, formic acid, and methanol. The hydroformylation approach poses environmental hazards through the use of rhodium- and nickel-based catalysts. In contrast, the thiol-ene reaction poses issues in scale and production efficiency as well as the environmental impact of the use of mercury lamps. Ozonolysis and transesterification technologies indicate difficulties with acid generation and toxicity hazards and require a detailed review of environmental impact and industrial feasibility assessment. Therefore, future research should concentrate on enhancing sustainability, reducing costs, and complying with environmental standards for wider use.

#### 3.1.2. Polyol Characterization

Exploratory studies on polyols derived from a variety of vegetable oils have underscored their potential applicability in adhesive formulation. The documented data (Table 3) divulges how varying material sources and extraction methodologies can engender polyols with disparate attributes.

The OHV, a significant parameter emblematic of a polyol’s reactivity and capability to form polymer networks, is pivotal. Despite OH groups’ integral role in polymer network formation and adhesive properties, overassertive OHV could catalyze excessive reactions and potentially disrupt adhesive quality via gas bubble creation [137,138,139]. Hence, the controlled moderation of OHVs is crucial to ensuring optimal performance.

The polyols isolated from sunflower and soybean oil through the epoxidation technique and from castor oil using the transesterification process display high OHV, implying a potential aptitude for participating in polymer network formation. However, high OHVs can also lead to high viscosity at room temperature and potentially catastrophic reactivity, warranting precise reaction management [139]. Lower-viscosity adhesives also demonstrate better substrate penetration, bolstering adhesive adhesion [140]. Thus, the operationality of such a polyol merits investigation.

Additionally, the acid value, indicative of free acid content, plays a crucial role in polyol characterization. Lower acid values denote minimal free acid content, thereby reducing the chance of interference with polymerization agents [45]. Polyols derived from camelia, linola flax, nulin flax, sunflower oil, and canola oil, using the epoxidation method, and castor oil through the thiol-ene coupling reaction method, demonstrated appropriate low acid values.

Finally, the polyol molecular weight (MW) is critical to the development of PUAs from vegetable oils because it directly impacts the resin’s characteristics and efficiency [141]. The optimal MW largely depends on the adhesive’s required properties: a lower MW for ease of processing and superior adhesion to yielding materials, or a higher MW for durability and strength. In light of a need for strength and longevity, higher MW options such as rubber seed oil processed via hydroformylation/hydrogenation, jatropha oil processed by epoxidation and hydroxylation, and castor oil processed via transesterification merit attention. Alternatively, in situations necessitating excellent processability and superior adhesion to flexible surfaces, the incorporation of lower MW ingredients, such as palm oil via epoxidation, canola oil via ozonolysis and hydrogenation, sunflower oil leveraging ozonolysis and hydrogenation, soybean oil using epoxidation, and camelia processed through ozonolysis and hydrogenation, may offer an optimal approach.

In summary, the utilization of polyols in adhesive production hinges on a careful selection of vegetable oil sources, extraction methods, and reasonable control of OHV, viscosity, acid value, and MW. All these factors profoundly impact the adhesive’s performance characteristics, indicating the importance of a thorough evaluation in the quest for high-quality, high-performance adhesive applications. This underlines the significant potential of vegetable oil-derived polyols in adhesive development, offering promising alternatives to traditional petroleum-based options with remarkable versatility and adaptability to specific performance needs.

### 3.2. Performance of Vegetable Oil-Based PUAs

#### 3.2.1. Isocyanate-Based PU

##### Exploring of PUAs

Researchers have extensively explored formulation techniques and additives to enhance vegetable oil-based PUAs. Among these techniques, a two-stage process involving pre-polymerization with diisocyanates like methylene diphenyl diisocyanate (MDI), toluene diisocyanate (TDI), and aliphatic diisocyanates (ADI), followed by cross-linking agent addition, offers flexibility in controlling molecular weight and cross-link density [41].

The progression of PUAs derived from sustainable sources has witnessed significant progress, with a multitude of investigations delving into the efficacy of utilizing diverse vegetable oils as substitute polyol reservoirs. Various vegetable oils that have undergone epoxidation, including canola, castor, palm, and jatropha oils, have taken the lead in these research endeavors. Several vegetable oil-based polyol and isocyanate formulations in the manufacture of PUAs are presented in Table 4. The properties of PUAs from various vegetable oil-based polyols that have been applied to the substrate are presented in Table 5.

**Figure 7:**
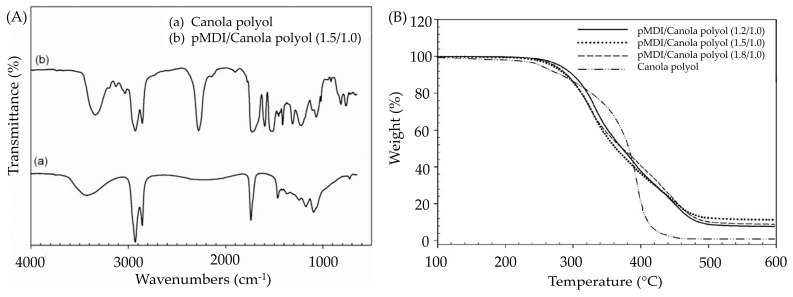
(**A**) FTIR spectra of canola oil polyol and canola oil-derived PU, (**B**) TGA curves of canola oil polyol and canola oil-derived PU with a different ratio of isocyanate groups to hydroxyl groups (RNCO:OH) (pMDI/canola polyol (1.2/1.0), pMDI/canola polyol (1.5/1.0) and pMDI/canola polyol (1.8/1.0)). Reprinted/adapted with permission from [31]. Elsevier, License Number (**A**) 5615251217866 (**B**) 5615251394743 2011.

Kong et al. [31] initiated the epoxidation of canola oil to produce polyols, which were subsequently reacted with polymeric methylene diphenyl diisocyanate (pMDI) to develop PUAs. Analysis of the chemical structure through FTIR spectroscopy confirmed the presence of urethane linkages at 3340 cm^−1^ for the OH group and at 1700 cm^−1^, thereby validating the successful formation of PUAs (Figure 7A). Furthermore, evaluations of thermal stability illustrated resilience up to 200 °C, suggesting their aptness for applications requiring high temperatures (Figure 7B). Based on this research, PUA with a RNCO:OH of 1.5/1.0 has the best results in lap shear strength of 5.7 MPa and glass transition temperature (Tg) of 101 °C. In terms of green strength, canola oil-based PU wood adhesive shows a lap shear strength increase of about 50% over the first three days before reaching a maximum. The canola oil-based PUAs demonstrated similar or better adhesive properties in terms of lap shear strength compared to the three commercial PUAs. The study also evaluated the effect of RNCO:OH and temperature on adhesive characteristics in wood bonding. It was found that using an elevated curing temperature (i.e., 100 °C) and an optimized RNCO:OH (higher than 1.5/1.0) improved the wood adhesive properties.

Research using palm oil-based polyols in PUA production has also been carried out. Ang et al. [98] focused on producing a polyester polyol using palm oil through an environmentally friendly method known as the ring-opening reaction. The synthesized polyol was combined with pMDI to explore further its potential applications and produce a high-performance PUA for wood bonding purposes. Remarkably, this adhesive demonstrated lap shear strength twice as strong as that of commercially available wood adhesives. Cui et al. [40] broadened the area of renewable resources by exploring crude glycerol, a byproduct of biodiesel production, as a viable feedstock for bio-polyol synthesis. Their pioneering study proved that the produced PUAs exhibit competitive lap shear strength and thermal stability, making crude glycerol a feasible choice for sustainable glue manufacture. The resulting PUA exhibited a strong lap shear strength of 36.8 MPa when a bio-polyol was reacted with isocyanate at RNCO:OH of 1.3, thus proving it as a robust and viable alternative.

Collectively, these distinct yet interconnected investigations offer a convincing story on the potential of plant-based polyols in the synthesis of isocyanate-based PUAs. The primary findings imply that by careful modification of the RNCO:OH and utilizing catalysts, the adhesive attributes such as lap shear strength, thermal stability, and curing characteristics can be greatly enhanced. Studies like those done by Somani et al. [34] have provided useful insights into how the choice of isocyanate adducts and the molecular weight of polyols influence the bonding capabilities and temperature resilience of the resulting adhesives. These polyols were subsequently combined with aromatic and aliphatic isocyanate adducts at varying RNCO:OH values ranging from 1.0 to 1.7, along with the addition of dibutyltin-dilaurate (DBTDL) catalyst at a concentration of 0.05 w/w%. Amongst these variables, an adhesive prepared with polyol A and aromatic isocyanate adduct at a RNCO:OH of 1.3 demonstrated superior adhesion strength and chemical resistance results.

A significant feature of PUA behavior rests in RNCO:OH. Tenorio-Alfonso et al. [18,19,36] undertook a series of investigations that proved the tremendous effect of this ratio on adhesive performance. By altering the RNCO:OH, they were able to regulate attributes such as bond strength and rigidity, finding that larger ratios often led to greater bond strength but at the potential cost of reduced flexibility. The results of the mechanical tests demonstrated that an increase in the RNCO:OH leads to higher adhesive hardness and, subsequently, increased shear strength values on both steel and wood substrates. Peel strength test results also revealed a gradual increase in strength values on wood as the RNCO:OH was raised to 4.53, with the highest values achieved at this specific ratio. Comparatively, PUA with RNCO:OH 4 and 4.53 exhibited peel strengths almost seven or eight times higher than those formulated with lower input ratios, such as PUA with RNCO:OH of 2. Similar trends were observed when stainless steel was used as the substrate for peel testing, wherein uniform peel strength values were obtained upon raising the functionalization level above 3.5, notably surpassing those attained on wood substrates.

Gama et al. [90] and Khoon et al. [41] noted the improved adhesion qualities of PUAs generated from castor and palm oil polyols when compared to commercial adhesives. The experiments indicate an ideal RNCO:OH that is important for increasing adhesive strength. Gama et al. [90] reported that free NCO (2270 cm^−1^) was detected in PUA with a RNCO:OH higher than one or an excess of isocyanate. Free NCO in RNCO:OH up to 2.50 can support adhesion strength on wood substrates because it can interact with OH groups. However, excessively high free NCO (RNCO:OH more than 2.50) can cause a decrease in adhesion strength, leading to adhesion failure. High free NCO values may lead to dimerization, which decreases the performance of the adhesive [142]. Free isocyanate content has a direct correlation to the shear strength of PUAs, as noted by Nacas et al. [142]. An increase in this content fortifies the shear strength. However, it is crucial to strike a balance, as an overabundance of free isocyanate may induce a process called dimerization, potentially undermining PUA properties. An upward shift in the RNCO:OH intensifies the rigidity of the PUA, pushing it towards a brittle state. This brittleness, in turn, risks compromising the adhesive strength. This observation has been echoed and v–alidated by Kong et al. [31], who reported excess isocyanate groups can instigate the production of unreacted isocyanates. These unreacted isocyanates are not only responsible for brittleness but also impair the adhesive strength. Additionally, Silva et al. [143] point out that a surfeit of isocyanate groups can foster crosslink formation, culminating in a more rigid and brittle adhesive. Such a state detracts from the adhesive flexibility and toughness, thereby degrading its bonding strength.

The breakthrough in bio-based PUAs was further proven by Du et al. [42], who produced a high-strength, self-healing hot-melt adhesive utilizing a symbiotic composition of vanillin oxime and soybean oil polyol. This unique formulation attained a large biomass content of 25% by weight, setting a new standard in the sector. After only 30 min of curing, the highest lap shear strength reached 6.55 ± 0.88 MPa. The presence of oxime–carbamate covalent bonds in the vegetable oil allowed for excellent self-healing capabilities and repeatable adhesion properties; even after seven complete breaking–repairing cycles, the lap shear strength remained at around 5.56 ± 0.89 MPa, surpassing more than 80% recovery when compared to initial performance levels. The Du et al. study was an exemplar for future research since their DPU adhesive displayed adaptability over multiple substrates, with excellent bonding strength and exceptional self-healing capabilities at low temperatures and in solvent-rich environments.

**Figure 8:**
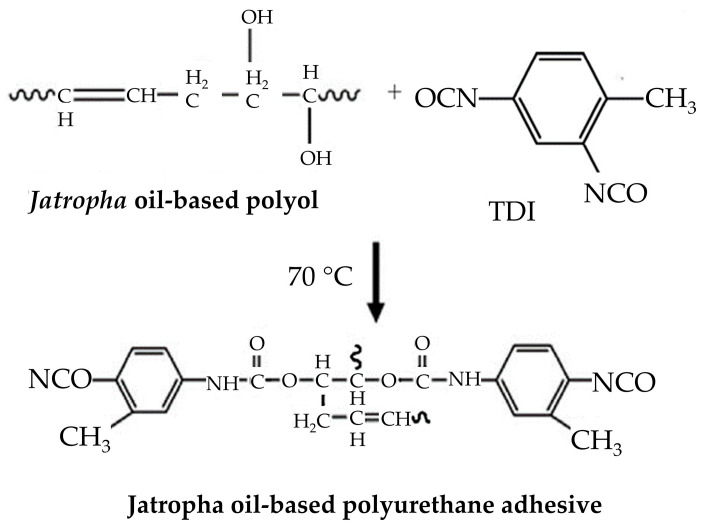
Hydroxylated polyol and prepolymer adhesive reaction with TDI [33]. Reprinted/adapted with permission from [33]. Elsevier, License Number 5615260382838 2014.

The environmental impact and functional efficacy of bio-based PUAs were also addressed by Aung et al. [33], who concentrated on the usage of jatropha oil. The reaction mechanism of TDI addition to jatropha oil is depicted in Figure 8. Varying the RNCO:OH within their formulations, they generated adhesives with substantial shear strength in both solid wood and plywood applications, exceeding palm oil-based competitors. This improved performance was related to higher cross-linking density at an RNCO:OH of 2.05:1.0, highlighting the promise of optimized bio-based adhesives in the woodworking sector.

Concomitantly, the environmental advantage of applying jatropha oil was not lost. Studies indicate that adhesives produced with this oil can potentially contribute to lower global warming potential, underlining the environmental stewardship component of this research. In addition to these specific experiments, Moghadam et al. [26] made important gains by creating PUAs for wood via the synthesis of polyester polyols from renewable sources, such as castor oil. Embracing green chemistry principles, they created adhesives with better water resistance and binding strength by co-polymerizing castor oil with different diacids. This work highlighted the trend towards not only renewable but also functional cross-linked structures appropriate for rigorous applications.

The aggregate findings from this modern research coalesce to produce a comprehensive understanding of how deliberate manipulation of RNCO:OH, as well as the integration of bio-based and renewable components, can result in improved PUA performance. The overall topic of sustainability is linked with functional performance, emphasizing that optimization is not just focused on physical properties but also the cultivation of environmentally responsible formulations.

Ultimately, the synthesis and refining of bio-based PUAs represent a robust convergence of innovation, sustainability, and performance. Moving forward, the field must continue to study the various paths of formulation optimization, including not just studies on the environmental footprint but also the lifecycle and longevity of these adhesive systems. The studies described herein serve as a launchpad for future investigations aiming at optimizing the delicate balance between these key aspects, opening the way for a new era of high-performance, sustainable adhesives.

##### Modification of Vegetable Oil-Based PUAs

The hunt for increased adhesion capabilities has led researchers to examine the strategic modification of PUAs. By merging the discoveries from the vanguard of material science, a unified story of innovation and advancement in adhesive modification emerges from recent investigations.

Malik and Kaur [32] highlighted how the incorporation of nanosized titanium dioxide as a filler into castor oil-based PUAs wrought considerable improvements in mechanical and chemical resistance, thermal glass transition temperature, and adhesion strength. The synthesis required mixing glycerol-modified castor oil with methylene diphenyl diisocyanate and adding titanium oxide (TiO_2_) nanoparticles, suggesting that fillers could take PUA performance to new heights. However, the integration of nanoparticles, such as TiO_2_, is not without environmental issues. The possible eco-toxicological implications of these nanoparticles, especially when released into agricultural areas, deserve careful attention and rigorous assessment. Simonin et al. have noted that TiO_2_ nanoparticles could severely affect soil microbial function, thereby disturbing critical ecological processes. Concurrently, Shi et al. [144] voiced concerns about the biodistribution and toxicity of TiO_2_ nanoparticles, underlining the necessity for a balanced strategy that includes both enhancement in adhesive qualities and their environmental repercussions.

A further breakthrough in adhesive modification was exhibited by Dodangeh et al. [35], who synthesized a bioadhesive from bio-polyol modified with epoxidized soybean oil and tetraethyl orthosilicate. The research focused on enhancing adhesive strength utilizing zinc oxide nanoparticles, triethylene glycol, and dibutyltin dilaurate additives. The optimization performed using the Taguchi approach suggested a great potential for soybean oil-based systems in PUA formulations.

Xu et al.’s recent study shed light on the thermally conductive structural adhesives utilizing castor oil-based PU and aluminum oxide (Al_2_O_3_) as a thermally conductive filler [29]. The formulation displayed outstanding tensile stress, elongation at break, and lap shear strength on varied substrates, emphasizing the adaptability of bio-based PUs when augmented with appropriate filler ingredients for increased thermal conductivity. It is obvious from this combined research that the pressing demand for sustainable yet high-performing adhesives has stimulated research into the infusion of fillers and modifiers in bio-based PU formulations. Adhesive performance is not merely enhanced through the cautious selection of basic polyols but also through the introduction of nanoparticles and other additives that contribute to the required mechanical, thermal, and adhesive qualities.

**Figure 9:**
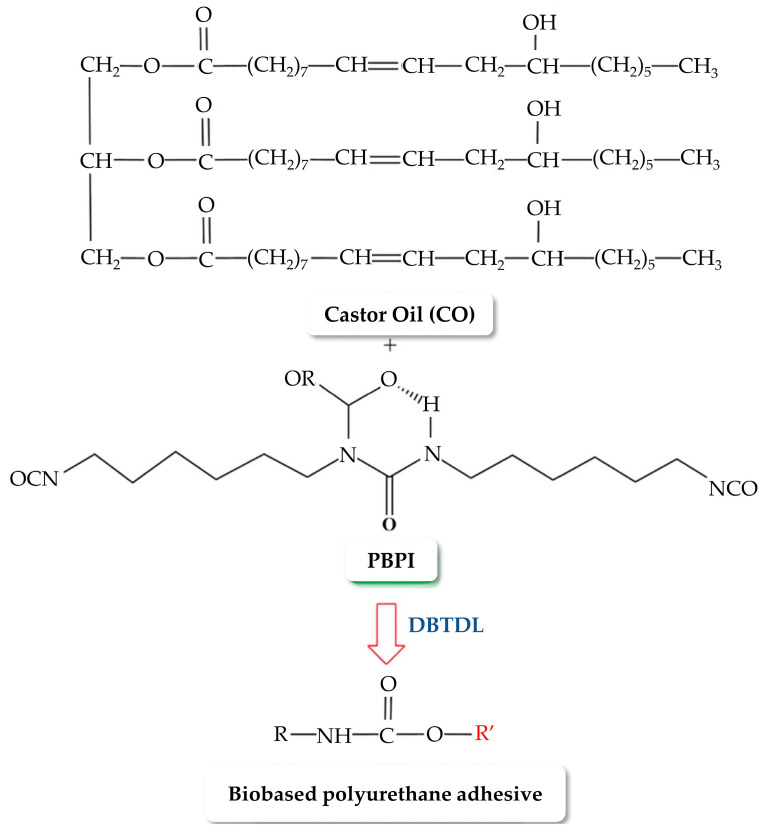
Reaction mechanism of bio-based PUA synthesis. DBTDL is dibutyltin-dilaurate, and R’ is Alkyl group. [21]. Reprinted/adapted with permission from [21]. Springer Nature, License Number 5616351351098 2017.

Sahoo et al. [21] dug into the synthesis of PU nanocomposite adhesives by utilizing trans-esterified castor oil-based polyol and organically modified montmorillonite nanoclay, displaying a notable elevation in lap shear strength when the RNCO:OH favored improved crosslinking. The mechanism underlying this process is illustrated in Figure 9. The introduction of nanoclay up to a threshold of 3 wt% was demonstrated to considerably boost adhesive strength, revealing an effective strategy for maximizing the bonding efficiency between substrates.

This collective discourse highlights a critical sentiment—bio-based PUAs can be fine-tuned to meet or exceed the performance limits of their synthetic competitors. The optimization approaches discussed not only show the rich tapestry of accessible modification techniques but also caution against the potential environmental implications that some of these adjustments may hold. It is this combined focus on enhancement and sustainability that must guide future research.

### 3.3. Non-Isocyanate-Based PU

In response to the mounting concerns surrounding the safety and environmental impact of traditional isocyanate-based PUs, the field of polymer science has undergone a paradigm shift towards non-isocyanate PUs. These next-generation materials offer a viable alternative, avoiding the toxicity and sensitivity difficulties connected with isocyanates, therefore spurring the creation of more sustainable and safer PUs. Non-isocyanate polyurethane (NIPU) is a safer and more promising PU than PU from isocyanates. NIPU networks are obtained by a synthesis route using diols, polyols, carbon dioxide, and diamines, which are far more environmentally friendly than isocyanates and phosgene [145]. With an increasing interest in NIPU coatings synthesized from vegetable oils, these substances are beginning to demonstrate desirable attributes for protective coverings and adhesives [146].

**Figure 10:**
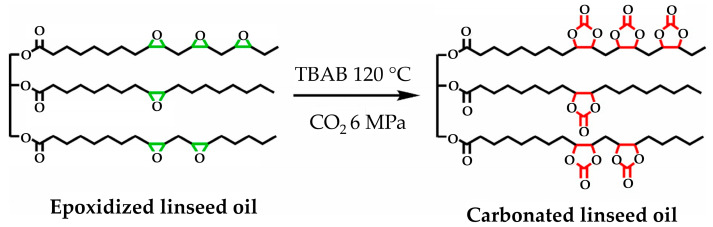
The scheme of CLSO synthesis from epoxidized linseed oil. The green color is epoxide group and the red color is carbonate group [27]. Reprinted/adapted with permission from [27]. Elsevier, License Number 5615240697632 2023.

**Figure 11:**
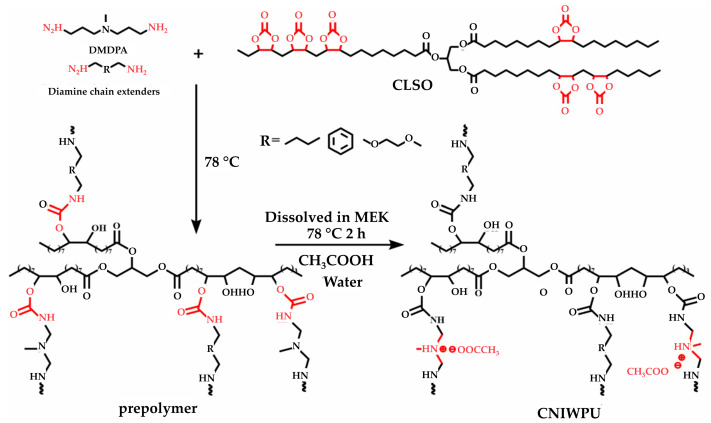
The scheme of cationic non-isocyanate waterborne poly(hydroxyl urethane)s (CNIWPU) synthesis [27]. Reprinted/adapted with permission from [27]. Elsevier, License Number 5615220027807 2023.

**Figure 12:**
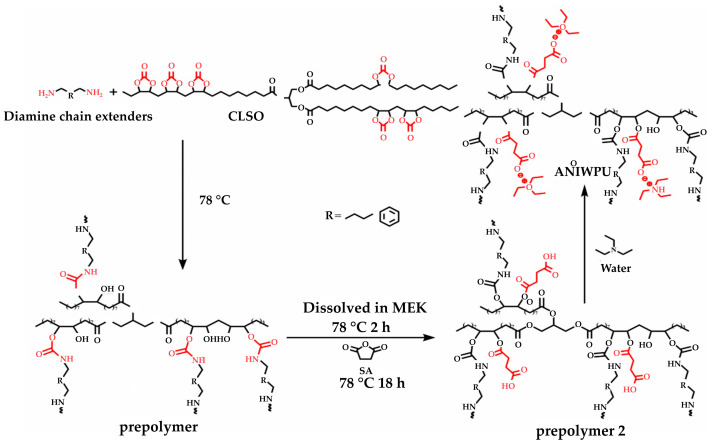
The scheme of anionic non-isocyanate waterborne poly(hydroxyl urethane)s (ANIWPU) synthesis [27]. Reprinted/adapted with permission from [27]. Elsevier, License Number 5615240842899 2023.

**Figure 13:**
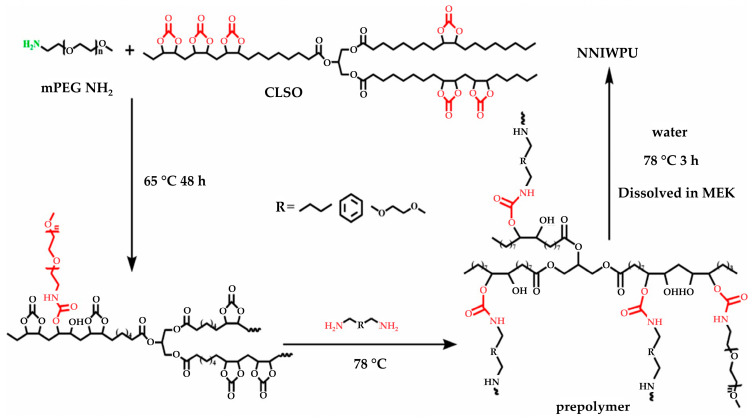
The scheme of non-ionic non-isocyanate waterborne poly(hydroxyl urethane)s (NNIWPU) synthesis [27]. Reprinted/adapted with permission from [27]. Elsevier, License Number 5615250169134 2023.

A summary of the literature demonstrates an emphasis on researching varied bio-based raw materials and creative paths to NIPU creation, highlighting the adaptability and environmental compatibility of these materials. Unverferth et al. [147] pioneered synthesis of NIPUs from castor oil and the exploitation of bio-based polyols which, through a polycondensation process employing dimethyl carbamate and diol with tri-n-butylamine as a catalyst, generate NIPUs without the use of hazardous isocyanates. Further confirming this change, Zhang et al.’s work on cationic, anionic, and nonionic waterborne NIPUs generated from carbonated linseed oil presents an eco-friendly approach [27]. Here, the epoxidized linseed oil is transmuted into carbonated linseed oil (CLSO) by interaction with carbon dioxide (Figure 10), establishing a carbon-neutral process. This CLSO is then polymerized by employing a range of diamines and hydrophilic moieties to create cationic, anionic, and non-ionic plant oil-based non-isocyanate waterborne polyurethane (NIWPU), as illustrated in Figure 11, Figure 12 and Figure 13. Their subsequent polymerization with diamines provides NIPUs with a range of desired features, including increased mechanical stability and considerable antibacterial effectiveness.

Delving into the mechanical integrity of NIPUs, the study of Doley and Dolui [122] indicates the potential of surface coating applications of NIPUs synthesized from carbonated sunflower oil and diamines. Their extensive investigation of changes in diamine structure and molar ratios serves as a testament to the stability of NIPUs for individual application requirements.

When critically appraised, these investigations jointly underline the developments in NIPU synthesis, underlining the crucial importance of both the source of raw materials and the synthetic methods. When critically appraised, these investigations jointly underline the developments in NIPU synthesis, underlining the crucial importance of both the source of raw materials and the synthetic methods. Through meticulous examination, researchers have not only exposed the inherent properties of NIPUs but also their prospective applications. By harnessing plant oils and employing benign reagents, studies have shown how NIPUs can approach or even surpass the performance of their isocyanate-based equivalents, notably in areas such as mechanical resilience, thermal stability, and adhesive qualities.

Moreover, the environmental and safety profiles of these materials have spurred heightened interest in their potential utility across a variety of industries, including the adhesive industry. The drive to integrate environmental sustainability with high-performance materials has never been more essential, as evidenced by society’s push towards green and clean technologies.

NIPUs represent this drive, with the potential to decrease carbon footprints, increase worker safety, and alleviate end-of-life environmental issues. Despite these significant gains, obstacles linger. The shift to NIPUs in industrial contexts needs careful consideration of scalability, cost competitiveness, and compatibility with current production infrastructures. Additionally, the long-term durability, weatherability, and robustness of NIPUs under varied application scenarios remain areas suitable for ongoing research.

#### Performance Evaluation

The evaluation of vegetable oil-based adhesives encompasses a multifaceted analysis of their bonding strength, durability, resistance to aging and environmental factors, moisture resistance, rheological properties, thermal behavior, chemical resistance, compatibility with different surfaces, and ecological sustainability. We provide an overview of the approaches used to analyze the performance of these adhesives and highlight the significant insights gained from previous research endeavors.

Evaluation of bonding strength often comprises standardized procedures, such as single lap shear joint testing or tension testing, as detailed in Table 3. Studies have proved the endurance and strength of vegetable oil-derived adhesives through accelerated aging experiments, including exposure to UV radiation or temperature cycling in humidity chambers. These tests imitate real-world conditions and provide useful insights into the adhesives’ robustness over time [46,139].

The efficacy of vegetable oil-based adhesives in resisting moisture is examined using various approaches, including water absorption tests following ASTM 570, water vapor permeance exploration as per ASTM E96/E96M, and water immersion testing. These evaluations measure changes in weight increase, size, and mechanical properties post-exposure to damp settings, providing vital data on the adhesives’ performance under wet conditions [33,74,139,148].

Assessment of rheological parameters, such as flow consistency, offers information about adhesive usability during application. Additionally, differential scanning calorimetry (DSC) and thermogravimetric analysis (TGA) are applied to study the adhesives’ behavior under varied temperatures, including Tg determination and decomposition rate analysis [149].

Chemical resistance examination analyzes the endurance of adhesives in various environmental circumstances, including exposure to water, acids, and alkaline solutions. Compatibility tests examine bonding efficacy with different surfaces and materials, ensuring optimal adhesive performance across broad applications.

Assessing the ecological sustainability of vegetable oil-based adhesives involves eco-toxicological study or LCA techniques. These evaluations gauge the environmental impact of adhesive production and consumption, supporting informed decision-making towards eco-friendly adhesive alternatives [109,110].

A thorough examination of available literature demonstrates the necessity for standardized testing methodologies to achieve uniform and trustworthy performance evaluations. Addressing inequalities and limits within current approaches is vital to improving the development of high-performance and ecologically conscious PUAs made from vegetable oils.

In conclusion, developing eco-friendly and sustainable adhesive alternatives requires standardized testing protocols and methodologies that account for influential elements and solve shortcomings within present practices. Implementing these findings can promote innovation and foster the creation of sturdy and environmentally friendly PUAs from renewable vegetable oil sources.

## 4. Conclusions

### 4.1. Summary of Findings

Several significant discoveries have been derived from this review article, which highlights the environmentally friendly, sustainable, and high-performance attributes of PUAs produced from vegetable oils. These findings are summarized below:

1. Vegetable oil offers a promising alternative to traditional petrochemical feedstocks owing to the abundance, renewability, and favorable chemical properties of adhesive mixtures.
2. The use of vegetable oils as renewable resources has shown promise in developing sustainable alternatives to PU components, replacing conventional materials while maintaining and even improving their adhesive properties.
3. The advancement of PUAs derived from renewable plants and tree sources represents a significant shift in research focus. This innovative approach holds great potential as it addresses the growing concern over the diminishing availability of fossil-based materials.
4. The growing popularity of vegetable oil-based adhesives as more environmentally friendly alternatives to petroleum-based materials has various advantages. This shift reduces the dependence on fossil fuels and significantly decreases the carbon footprint associated with the use and production of these adhesives.

In summary, using vegetable oils in the production of PUAs offers a promising solution for achieving sustainability and reducing environmental impact compared to conventional petroleum-based materials.

### 4.2. Recommendations for Further Research

In light of this review, several potential areas of study have been identified, which could significantly advance the existing knowledge and application of eco-friendly, sustainable, and high-performing vegetable oil-based PUAs:

1. Diversification of Vegetable Oil Sources: A promising area of research involves the examination of various vegetable oils. Such oils range from canola and corn oil to more unconventional sources like rubber seed oil, crude algal oil, sunflower oil, camelia oil, and jatropha oil. The primary aim was to identify the most suitable raw materials for adhesive formulations.
2. Creation of Bio-based Isocyanates: Research addressing the current limitations of employing non-renewable isocyanates in bio-based PUAs can reconceptualize adhesive construction. This could entail the development of bio-based isocyanates, which would effectively increase the overall renewable content of PU.
3. Tuning of Adhesive Properties: Investigating how formulation techniques, raw materials, and employed additives can better optimize performance characteristics, such as bonding strength, durability, and environmental resistance, is another potential area of focus.
4. Discovery of Non-Isocyanate-Based PUAs: A deeper study into the possible use of alternative raw materials, such as amines, instead of isocyanates may open new doors for developing non-isocyanate-based PUAs.
5. Assessment of Biodegradability: A critical aspect of ensuring minimal environmental impact involves evaluating the biodegradability of vegetable oil-based PUAs.
6. Navigating Regulatory Compliance and Industry Adoption: Lastly, addressing challenges related to meeting regulatory requisites and promoting the industry adoption of such adhesives is vital for broad-spectrum usage in sectors including construction, automotive, packaging, and textiles.

By filling in these research gaps, future studies can significantly contribute towards establishing more sustainable and environmentally friendly PUAs derived from vegetable oils.

## Figures and Tables

**Table 1:** Comparison of adhesives by category.

Adhesive Class	Raw Material	Main Advantages	Main Challenges	Main Applications	Ref.
Protein adhesives	Plant and animal proteins (casein, collagen, fish protein, vegetable protein)	Environmentally friendly, flexible in various applications	Low water resistance, susceptible to degradation due to extreme environments	Wood and paper-making industry, wood paneling, furniture	[60]
Rubber-based adhesives	Natural or synthetic rubber latex	High elasticity and flexibility	Low heat resistance	Automotive industry, packaging, electronics, shoe and clothing makers, and construction sector	[61]
Phenolic resin adhesives	Phenol, formaldehyde	High heat resistance	Environmental impact, limitations in application (less suitable for applications that require elastic adhesion)	Wood adhesives, laminates, construction	[62]
Resorcinol and phenol-resorcinol adhesives	Resorcinol, phenol, formaldehyde	High reactivity (room-temperature curing), good storage stability, strong adhesion, water resistance	High cost of resorcinol	Exterior-grade adhesives, structural application area, laminates, construction	[62]
Natural phenolic adhesives	Tannin, lignin	High biodegradability, environmentally friendly	Limited adhesion properties	Wood panel, paper, and cardboard industry	[63]
Urea and melamine amino resin adhesives	Urea, melamine, formaldehyde	Resistant to moisture, good storage stability, strong adhesion	Does not withstand exterior applications, potential formaldehyde emissions	Particle and fiberboard, lining paper, lamination, furniture and decoration industries	[63]
PUA	Polyols and isocyanates	Strength and durability, adhesion to various substrates	Toxicity, environmental impact	Automotive, construction, wood and furniture, packaging, and textile industries	[64]
Reactive acrylic adhesives	Acrylic polymer	Strength, temperature, and chemical resistance	Lower rigidity than other structural adhesives	Automotive adhesives, construction, general manufacturing	[65]
Anaerobic adhesives	Methacrylate	Fills gaps and cracks, vibration resistance, corrosion protection	Clean surface dependency	Thread locking, retaining, thread sealing, general industry applications	[66]
Aerobic acrylic adhesives	Oligomer dan monomer methacrylate	Adhesion to polyolefins, resistance to temperature and chemicals	Some adhesives suffer from oxygen inhibition, so they remain tacky on surfaces, incompatibility with specific polymers	Connection of magnets, displays, and medical needles	[67]
Biobased acrylic adhesives	Acrylic acid, methyl methacrylate, and other (meth)acrylate monomers	Environmentally friendly	High production cost	Automotive industry	[68]
Silicone adhesives and sealants	Polydimethylsiloxane (PDMS) polymer	Resistance to temperature and chemicals, resistance to ultraviolet (UV) radiation	High production cost, slow curing time	Construction, automotive, electronics, specialty applications	[69]
Epoxy adhesives	Epichlorohydrin and bis-phenol-A	Strength, temperature, and chemical resistance	Relatively expensive, not resistant to UV light, low resistance to organic fertilizers	Construction, electronics, automotive industry adhesives	[70]
Bio-sourced epoxy monomers and polymers	Epoxy based on natural materials (vegetable oils, lignin)	Good chemical resistance, high-temperature resistance, flexibility	Low water resistance	Construction, automotive	[71]
Pressure-sensitive adhesive	Elastomers, visco-elastomers, tackifier resins, plasticizers, etc.	Ease of use, easy to remove	Lower strength compared to other adhesives	Adhesive labels, stickers, tapes, protective films of electronic surfaces, glass, or other products	[72]

**Table 2:** Comparison of polyol extraction methods.

Polyol Extraction Method	Advantages	Challenges	Potential Developments	Ref.
Epoxidation/Oxirane Ring Opening	Vegetable oils are a renewable resource with environmental sustainability potential. They produce polyols with moderate hydroxyl values.	Chemical usage, such as hydrogen peroxide and formic acid, can lead to pollution risks. Methanol has the potential to impact air quality negatively.	Further research is necessary to reduce the use of dangerous chemicals and discover environmentally friendly options.	[52,98]
Hydroformylation	Utilizes rubber seed oils, reduces dependence on fossil fuels, and optimizes waste management effectively.	It relies on expensive rhodium and nickel catalysts, causing water pollution due to nickel.	Further investigation is required for environmentally friendly catalysts and optimizing processes to reduce environmental footprint.	[30,127]
Thiol-ene Reaction	Utilizes renewable biomass to create high OH-value (OHV) polyol.	Challenges involve low temperature, extended duration, and scale-up difficulties.	Further research is needed to discover new reactants, enhance conditions, and boost efficiency in time and energy.	[13,16]
Ozonolysis	Facilitates the production of high OHV polyol and promotes the recycling of vegetable oil for renewable feedstock.	Acid formation during ozonolysis can hinder polyurethane (PU) formation. The environmental advantages of crude oil utilization need a more comprehensive assessment.	Optimize to reduce acid formation, compare environmental impact with traditional methods, and reduce total cost by recycling unused ozone.	[94,124]
Transesterification	Utilizes vegetable oils for renewable feedstock, offering improved control over polyol characteristics and producing polyols with varied OHV.	Necessitates elevated reaction temperature and prolonged reaction time, as well as the utilization of possibly contaminating catalysts.	Additional research is necessary to enhance reaction conditions and discover environmentally friendly catalysts.	[22,23,133]

**Table 3:** Characteristics of polyols prepared from different vegetable oil sources and extraction methods.

Raw Mat.	Method	OHV ^1^	AV ^2^	MW ^3^	Viscosity ^4^	Ref.
Used Palm Cooking Oil	Ozonolysis	85–202	-	-	0.015	[94]
Palm oil	Epoxidation	78.17	2.74	36.308	0.041	[41]
	Epoxidized and hydroxylation	132	7.56	922	85	[33]
Canola oil	Ozonolysis	-	-	680–1066	-	[125]
	Ozonolysis and hydrogenation	203	-	521	-	[17]
	Ozonolysis	260	2	-	0.81	[14]
	Epoxidation and ring-opened	259	0,2	-	3.584 × 10^6^	[31]
	Epoxidation	259	0.8	-	2.4	[135]
	Esterification	164.6	-	-	-	[90]
Corn Oil	Ozonolysis	163.1	-	-	-	[126]
	Epoxidation	140.8	-	-	-	[126]
Crude Algal Oil	Ozonolysis	123	-	-	-	[127]
	Epoxidation/ ring opening	51.6	13.7	-	470	[127]
	Transesterific-ation	150	3.3	-	1.3	[127]
	Hydroformyl-ation	147	-	-	-	[127]
Rubber Seed Oil	Hydroformyl-ation/Hydrogen-ation	240–244	21	1900	10.6	[30]
Sunflower Oil	Ozonolysis and hydrogenation	210	11	563	0.5	[124]
	Epoxidation/ ring opening	402	-	-	-	[136]
	Thiol-ene coupling reaction	-	-	-	-	[136]
	Epoxidation	286	0.6	-	3.3	[135]
Soybean Oil	Ozonolysis	228	2	-	0.68	[14]
	Ozonolysis	-	-	-	-	[15]
	Thiol-ene coupling reaction	199	-	-	-	[13]
	Thiol-ene coupling reaction	203	-	1270	-	[16]
	Epoxidation	300	-	-	-	[11]
	Epoxidation ring opening	158–283	2.73–4.56	501–13.615	37.5–688	[17]
Jatropha Oil	Epoxidation and hydroxylation	171–179	10.4–12.2	1681–1710	0.92–0.98	[128]
	Epoxidation/oxarine ring opening	138–217	4.80–8.63	-	30.0–80.4	[129]
	Hydroxylation and alcoholysis	171	8.19	1251	75	[33]
Castor Oil	Transesterific-ation	190–234	1.40–1.68	3490–3931	0.98–0.99	[22]
	Transesterific-ation	160–250	2	-	-	[23]
	Thiol-ene coupling reaction	278	-	1167	-	[16]
	Thiol-ene coupling reaction	258–286	0.98–2.74	-	15.5–18.6	[24]
	Transesterific-ation	350–450	-	1165	-	[133]
	Esterification	117–134	1.41–1.65	-	0.62–0.945	[26]
Crude Alga Oil	Hydroformyl-ation	147	-	-	-	[127]
	Ozonolysis	123	-	-	-	[127]
	Epoxidation	51.6	-	-	0.47	[127]
	Transesterific-ation	150	3.3	-	1.3	[127]
Camelia	Ozonolysis and hydrogenation	165	7	692	0.4	[124]
	Epoxidation	272	0.5	-	4.7	[135]
Linola flax	Epoxidation	292	0.9	-	4.2	[135]
Nulin Flax	Epoxidation	302	0.8	-	13.5	[135]
Sunflower	Epoxidation	-	-	876.19	-	[122]

^1^ OHV: OH value (mg KOH g^−1^), ^2^ AV: acid value (mg KOH g^−1^), ^3^ MW: molecular weight (g mol^−1^), ^4^ viscosity at room temperature (Pa∙s).

**Table 4:** The PUA formulas on various polyols and isocyanates.

No	Polyol	Isocyanate	RNCO:OH	Ref.
1	Epoxidated canola oil	pMDI	1.2/1.0, 1.5/1.0, 1.8/1.0.	[31]
2	Glycerol modified castor oil	MDI	1.0–1.4	[32]
3	Esterified castor oil	MDI	1.0–1.6	[26]
4	Palm oil polyester polyol	pMDI, TDI	1.3, 1.5	[98]
5	Jatropha oil-based polyol	TDI	1.8/1.0 2.05/1.0 2.2/1.0	[33]
6	Palm oil-based polyol	TDI	1.8/1.0 2.05/1.0 2.2/1.0	[33]
7	Castor oil	MDI	1.00–3.00	[90]
8	Castor oil polyester polyol	Aromatic and aliphatic isocyanate	1.0, 1.3, 1.7	[34]
9	Soybean oil polyol	IPDI	1.05	[42]
10	Epoxidated soybean oil	pMDI	3/2	[35]
11	Castor oil	PBPI	1.1:1, 1.3:1, and 1.5:1	[21]
12	Castor oil	HMDI	1.87 & 3.20	[19]
13	Castor oil polyol	PAPI	Under 1.1.5	[29]
14	Crude glycerol	MDI	1.0 to 1.7	[107]
15	Castor oil	MDI	2:1, 2.5:1, 3:1, 3.5:1, 4:1, 4.53:1	[18]
16	Castor oil	HMDI	4.53:1:1	[36]

**Table 5:** Properties of PUAs on various polyols and isocyanates.

No	Bio-PU adhesive	Application	LSS ^1^ *	LF ^2^ *	CT ^3^ *	GS ^4^ *	Ref.
1	Epoxidated canola oil	Wood	5.7	CF+AF+SF	3	N/A	[31]
2	Glycerol modified castor oil	Wood	39–46	N/A	4	51	[32]
3	Esterified castor oil	Wood	20–35	N/A	7	24–35	[26]
4	Palm oil polyester	Solid wood	5.3	SF	5	5.2	[98]
5	Jatropha oil-based polyol	Wood	3.5–3.9	CF+AF	N/A	N/A	[33]
6	Palm oil-based polyol	Wood	1.5–1.9	N/A	N/A	N/A	[33]
7	Castor oil	Wood	0.01–1.80	N/A	3	1.89	[90]
8	Castor oil polyester polyol	Wood	56.3–96.9	N/A	7	N/A	[34]
9	Soybean oil polyol	Carbon steel, aluminum, poplar, and wood–plastic composite	2.14–6.55	N/A	N/A	N/A	[42]
10	Epoxidated soybean oil	Wood	5.04–5.22	SF	N/A	N/A	[35]
11	Castor oil	Wood	19 × 10^−5^–40 × 10^−5^	N/A	30	N/A	[21]
12	Castor oil	Chips and metal	3.773–4.422	N/A	N/A	N/A	[29]
13	Crude glycerol	Wood	36.8 ± 2.5	N/A	4	N/A	[40]

^1^ Lap shear strength (MPa), ^2^ locus of failure, ^3^ curing time (day), ^4^ green strength (MPa). * CF = cohesion failure, AF = adhesion failure, SF = surface failure.

## Data Availability

Not applicable.

## References

[1] Awad J., Jung C. (2021). Evaluating the Indoor Air Quality after Renovation at the Greens in Dubai, United Arab Emirates. Buildings.

[2] Van Tran V., Park D., Lee Y.C. (2020). Indoor Air Pollution, Related Human Diseases, and Recent Trends in the Control and Improvement of Indoor Air Quality. Int. J. Environ. Res. Public Health.

[3] Statista (2024). Annual Volatile Organic Compounds (VOC) Emissions in the United States from 1970 to 2023.

[4] WHO (2010). WHO Guidelines for Indoor Air Quality: Selected Pollutants.

[5] Bernstein J.A., Alexis N., Bacchus H., Bernstein I.L., Fritz P., Horner E., Li N., Mason S., Nel A., Oullette J. (2008). The Health Effects of Nonindustrial Indoor Air Pollution. J. Allergy Clin. Immunol..

[6] Brickus L.S.R., Cardoso J.N., De Aquino Neto F.R. (1998). Distributions of Indoor and Outdoor Air Pollutants in Rio de Janeiro, Brazil: Implications to Indoor Air Quality in Bayside Offices. Environ. Sci. Technol..

[7] Ghaffarianhoseini A., Al Waer H., Omrany H., Ghaffarianhoseini A., Alalouch C., Clements-Croome D., Tookey J. (2018). Sick Building Syndrome: Are We Doing Enough?. Archit. Sci. Rev..

[8] Gogoi S., Karak N. (2014). Biobased Biodegradable Waterborne Hyperbranched Polyurethane as an Ecofriendly Sustainable Material. ACS Sustain. Chem. Eng..

[9] Blasco M.P.C., Limiñana M.Á.P., Silvestre C.R., Calpena E.O., Aís F.A. (2022). Sustainable Reactive Polyurethane Hot Melt Adhesives Based on Vegetable Polyols for Footwear Industry. Polymers.

[10] Gadhave R.V., Mahanwar P.A., Gadekar P.T. (2017). Bio-Renewable Sources for Synthesis of Eco-Friendly Polyurethane Adhesives—Review. Open J. Polym. Chem..

[11] Acik G., Kamaci M., Altinkok C., Karabulut H.R.F., Tasdelen M.A. (2018). Synthesis and Properties of Soybean Oil-Based Biodegradable Polyurethane Films. Prog. Org. Coat..

[12] Zhang C., Xia Y., Huh S., Johnston P.A., Kessler M.R. (2013). Soy-Castor Oil Based Polyols Prepared Using a Solvent-Free and Catalyst-Free Method and Polyurethanes Therefrom. Green.

[13] Feng Y., Yang Z., Liang H., Yang Z., Yuan T., Luo Y., Li P., Zhang C. (2017). A Solvent-Free and Scalable Method to Prepare Soybean-Oil-Based Polyols by Thiol-Ene Photo-Click Reaction and Biobased Polyurethanes Therefrom. ACS Sustain. Chem. Eng..

[14] Petrović Z.S., Zhang W., Javni I. (2005). Structure and Properties of Polyurethanes Prepared from Triglyceride Polyols by Ozonolysis. Biomacromolecules.

[15] Tran P., Graiver D., Narayan R. (2005). Ozone-Mediated Polyol Synthesis from Soybean Oil Phuong. J. Am. Oil Chem. Soc..

[16] Alagi P., Choi Y.J., Seog J., Hong S.C. (2016). Efficient and Quantitative Chemical Transformation of Vegetable Oils to Polyols through a Thiol-Ene Reaction for Thermoplastic Polyurethanes. Ind. Crops Prod..

[17] Karadeniz K., Çalıkoğlu Y., Sen M.Y. (2017). A Novel Polyurethanes from Epoxidized Soybean Oil Synthesized by Ring Opening with Bifunctional Compounds. Polym. Bull..

[18] Tenorio-Alfonso A., Sánchez M.C., Franco J.M. (2019). Synthesis and Mechanical Properties of Bio-Sourced Polyurethane Adhesives Obtained from Castor Oil and MDI-Modified Cellulose Acetate: Influence of Cellulose Acetate Modification. Int. J. Adhes. Adhes..

[19] Tenorio-Alfonso A., Sánchez M.C., Franco J.M. (2022). Impact of the Processing Method on the Properties of Castor Oil/Cellulose Acetate Polyurethane Adhesives for Bonding Wood. Int. J. Adhes. Adhes..

[20] Pathak R., Kathalewar M., Wazarkar K., Sabnis A. (2015). Non-Isocyanate Polyurethane (NIPU) from Tris-2-Hydroxy Ethyl Isocyanurate Modified Fatty Acid for Coating Applications. Prog. Org. Coat..

[21] Sahoo S., Kalita H., Mohanty S., Nayak S.K. (2017). Synthesis and Characterization of Vegetable Oil Based Polyurethane Derived from Low Viscous Bio Aliphatic Isocyanate: Adhesion Strength to Wood-Wood Substrate Bonding. Macromol. Res..

[22] Das S., Pandey P., Mohanty S., Nayak S.K. (2015). Influence of NCO/OH and Transesterified Castor Oil on the Structure and Properties of Polyurethane: Synthesis and Characterization. Mater. Express.

[23] Valero M.F., Gonzalez A. (2012). Polyurethane Adhesive System from Castor Oil Modified by a Transesterification Reaction. J. Elastomers Plast..

[24] Ionescu M., Radojčić D., Wan X., Shrestha M.L., Petrović Z.S., Upshaw T.A. (2016). Highly Functional Polyols from Castor Oil for Rigid Polyurethanes. Eur. Polym. J..

[25] Agrawal A., Kaur R., Singh Walia R. (2019). Flame Retardancy of Ceramic-Based Rigid Polyurethane Foam Composites. J. Appl. Polym. Sci..

[26] Moghadam P.N., Yarmohamadi M., Hasanzadeh R., Nuri S. (2016). Preparation of Polyurethane Wood Adhesives by Polyols Formulated with Polyester Polyols Based on Castor Oil. Int. J. Adhes. Adhes..

[27] Zhang W., Wang T., Zheng Z., Quirino R.L., Xie F., Li Y., Zhang C. (2023). Plant Oil-Based Non-Isocyanate Waterborne Poly(Hydroxyl Urethane)S. Chem. Eng. J..

[28] Bähr M., Mülhaupt R. (2012). Linseed and Soybean Oil-Based Polyurethanes Prepared via the Non-Isocyanate Route and Catalytic Carbon Dioxide Conversion. Green Chem..

[29] Xu C., Jia X., Du J., Zhou F., Liu B., Deng Y., Huai X. (2023). Ultra-Strong and Solvent-Free Castor Oil-Based Polyurethane Thermally Conductive Structural Adhesives for Heat Management. Ind. Crops Prod..

[30] Hong J., Radojčić D., Yang X.Q., Wan X., Petrović Z.S. (2019). Tough Thermosetting Polyurethanes and Adhesives from Rubber Seed Oil by Hydroformylation. J. Appl. Polym. Sci..

[31] Kong X., Liu G., Curtis J.M. (2011). Characterization of Canola Oil Based Polyurethane Wood Adhesives. Int. J. Adhes. Adhes..

[32] Malik M., Kaur R. (2018). Mechanical and Thermal Properties of Castor Oil–Based Polyurethane Adhesive: Effect of TiO_2_ Filler. Adv. Polym. Technol..

[33] Aung M.M., Yaakob Z., Kamarudin S., Abdullah L.C. (2014). Synthesis and Characterization of Jatropha (*Jatropha curcas* L.) Oil-Based Polyurethane Wood Adhesive. Ind. Crops Prod..

[34] Somani K.P., Kansara S.S., Patel N.K., Rakshit A.K. (2003). Castor Oil Based Polyurethane Adhesives for Wood-to-Wood Bonding. Int. J. Adhes. Adhes..

[35] Dodangeh F., Seyed Dorraji M.S., Rasoulifard M.H., Ashjari H.R. (2020). Synthesis and Characterization of Alkoxy Silane Modified Polyurethane Wood Adhesive Based on Epoxidized Soybean Oil Polyester Polyol. Compos. Part B Eng..

[36] Tenorio-Alfonso A., Sánchez M.C., Franco J.M. (2017). Preparation, Characterization and Mechanical Properties of Bio-Based Polyurethane Adhesives from Isocyanate-Functionalized Cellulose Acetate and Castor Oil for Bonding Wood. Polymers.

[37] Chen Q., Xu X., Zhang X., Xu Z., Liu Y., Huan S., Li Z., Bai L., Gu J. (2023). Valorization of Isocyanates Using Castor Oil-Based Protective Strategies: Performance and Comparison as Waterborne Adhesive Additives. Ind. Crops Prod..

[38] Liang H., Feng Y., Lu J., Liu L., Yang Z., Luo Y., Zhang Y., Zhang C. (2018). Bio-Based Cationic Waterborne Polyurethanes Dispersions Prepared from Different Vegetable Oils. Ind. Crops Prod..

[39] Gama N.V., Ferreira A., Barros-Timmons A. (2018). Polyurethane Foams: Past, Present, and Future. Materials.

[40] Cui S., Liu Z., Li Y. (2017). Bio-Polyols Synthesized from Crude Glycerol and Applications on Polyurethane Wood Adhesives. Ind. Crops Prod..

[41] Ang K.P., Lee C.S., Cheng S.F., Chuah C.H. (2014). Polyurethane Wood Adhesive from Palm Oil-Based Polyester Polyol. J. Adhes. Sci. Technol..

[42] Du L., Liu Z., Ye Z., Hao X., Ou R., Liu T., Wang Q. (2023). Dynamic Cross-Linked Polyurethane Hot-Melt Adhesive with High Biomass Content and High Adhesive Strength Simultaneously. Eur. Polym. J..

[43] Erickson B., Nelson J.E., Winters P. (2012). Perspective on Opportunities in Industrial Biotechnology in Renewable Chemicals. Biotechnol. J..

[44] Kemona A., Piotrowska M. (2020). Polyurethane Recycling and Disposal: Methods and Prospects. Polymers.

[45] Gomez J.C., Zakaria R., Aung M.M., Mokhtar M.N., Yunus R. (2021). Synthesis and Characterization of Polyurethanes from Residual Palm Oil with High Poly-Unsaturated Fatty Acid Oils as Additive. Polymers.

[46] Fiorelli J., Curtolo D.D., Barrero N.G., Savastano H., de Jesus Agnolon Pallone E.M., Johnson R. (2012). Particulate Composite Based on Coconut Fiber and Castor Oil Polyurethane Adhesive: An Eco-Efficient Product. Ind. Crops Prod..

[47] Zaia U.J., Cortez-Barbosa J., Morales E.A.M., Lahr F.A.R., Do Nascimento M.F., De Araujo V.A. (2015). Production of Particleboards with Bamboo (*Dendrocalamus giganteus*) Reinforcement. BioResources.

[48] Cravo J.C.M., de Lucca Sartori D., Mármol G., Schmidt G.M., de Carvalho Balieiro J.C., Fiorelli J. (2017). Effect of Density and Resin on the Mechanical, Physical and Thermal Performance of Particleboards Based on Cement Packaging. Constr. Build. Mater..

[49] Gava M., Müzel S.D., de Lima L.R., Cortez-Barbosa J., Garcia J.N., Ferreira B.S., Filho H.J.S., Bernardes M.S., De Araujo V.A. (2015). Production of Particleboards from Hevea Brasiliensis Clones and Castor Oil-Based Polyurethane Resin. BioResources.

[50] Sugahara E.S., da Silva S.A.M., Buzo A.L.S.C., de Campos C.I., Morales E.A.M., Ferreira B.S., dos Anjos Azambuja M., Lahr F.A.R., Christoforo A.L. (2019). High-Density Particleboard Made from Agro-Industrial Waste and Different Adhesives. BioResources.

[51] Zain N.M., Roslin E.N., Ahmad S. (2016). Preliminary Study on Bio-Based Polyurethane Adhesive/Aluminum Laminated Composites for Automotive Applications. Int. J. Adhes. Adhes..

[52] Mekewi M.A., Ramadan A.M., ElDarse F.M., Abdel Rehim M.H., Mosa N.A., Ibrahim M.A. (2017). Preparation and Characterization of Polyurethane Plasticizer for Flexible Packaging Applications: Natural Oils Affirmed Access. Egypt. J. Pet..

[53] Sidra, Tabasum S., Zia K.M., Parveen B., Hussain M.T. (2021). A Novel Water Borne Green Textile Polyurethane Dispersions Finishes from Cotton (*Gossypium arboreum*) Seed Oil Based Polyol Used in Modification of Cellulosic Fabrics. Carbohydr. Polym. Technol. Appl..

[54] Santan H.D., James C., Fratini E., Martínez I., Valencia C., Sánchez M.C., Franco J.M. (2018). Structure-Property Relationships in Solvent Free Adhesives Derived from Castor Oil. Ind. Crops Prod..

[55] De Ponte C., Liscio M.C., Sospiro P. (2023). State of the Art on the Nexus between Sustainability, Fashion Industry and Sustainable Business Model. Sustain. Chem. Pharm..

[56] Orgilés-Calpena E., Arán-Aís F., Torró-Palau A.M., Orgilés-Barceló C. (2016). Novel Polyurethane Reactive Hot Melt Adhesives Based on Polycarbonate Polyols Derived from CO2 for the Footwear Industry. Int. J. Adhes. Adhes..

[57] Norazwani M.Z., Ghazali F.A., Roslin E.N. (2018). Potential of Natural Oil-Based Polyurethane as an Adhesive for Particleboard Production: A Review. Int. J. Mech. Eng. Technol..

[58] Zhang H., Guo Y., Yao J., He M. (2016). Epoxidised Soybean Oil Polymer Composites Reinforced with Modified Microcrystalline Cellulose. J. Exp. Nanosci..

[59] Pizzi A., Mittal K.L. (2018). Handbook of Adhesive Technology.

[60] Pizzi A., Mittal K.L., Frihart C.R., Lorenz L.F. (2018). Protein Adhesives. Handbook of Adhesive Technology.

[61] Pizzi A., Mittal K.L., Shybi A.A., Varghese S., Maria H.J., Thomas S. (2018). Rubber-Based Adhesives. Handbook of Adhesive Technology.

[62] Pizzi A., Mittal K.L., Pizzi A. (2018). Phenolic Resin Adhesives. Handbook of Adhesive Technology.

[63] Pizzi A., Mittal K.L., Pizzi A. (2018). Natural Phenolic Adhesives Derived from Tannins and Lignin. Handbook of Adhesive Technology.

[64] Pizzi A., Mittal K.L., Lay D.G., Cranley P., Pizzi A. (2018). Polyurethane Adhesives. Handbook of Adhesive Technology.

[65] Pizzi A., Mittal K.L., Pitia E., Hill J. (2018). Reactive Acrylic Adhesives. Handbook of Adhesive Technology.

[66] Pizzi A., Mittal K.L., Birkett D., Condron D. (2018). Anaerobic Adhesives. Handbook of Adhesive Technology.

[67] Pizzi A., Mittal K.L., Sweeney N. (2018). Aerobic Acrylic Adhesives. Handbook of Adhesive Technology.

[68] Pizzi A., Mittal K.L., Sweeney N. (2018). Biobased Acrylic Adhesives. Handbook of Adhesive Technology.

[69] Pizzi A., Mittal K.L., Klosowski J.M. (2018). Silicone Adhesives and Sealants. Handbook of Adhesive Technology.

[70] Pizzi A., Mittal K.L., Rudawska A. (2018). Epoxy Adhesives. Handbook of Adhesive Technology.

[71] Pizzi A., Mittal K.L., Caillol S., Boutevin B., Pascault J.-P. (2018). Bio-Sourced Epoxy Monomers and Polymers. Handbook of Adhesive Technology.

[72] Pizzi A., Mittal K.L., Benedek I. (2018). Pressure-Sensitive Adhesives. Handbook of Adhesive Technology.

[73] Ghasemlou M., Daver F., Ivanova E.P., Adhikari B. (2019). Polyurethanes from Seed Oil-Based Polyols: A Review of Synthesis, Mechanical and Thermal Properties. Ind. Crops Prod..

[74] Liang H., Liu L., Lu J., Chen M., Zhang C. (2018). Castor Oil-Based Cationic Waterborne Polyurethane Dispersions: Storage Stability, Thermo-Physical Properties and Antibacterial Properties. Ind. Crops Prod..

[75] Handika S.O., Lubis M.A.R., Sari R.K., Laksana R.P.B., Antov P., Savov V., Gajtanska M., Iswanto A.H. (2021). Enhancing Thermal and Mechanical Properties of Ramie Fiber via Impregnation by Lignin-Based Polyurethane Resin. Materials.

[76] Iswanto A.H., Lubis M.A.R., Sutiawan J., Al-Edrus S.S.O., Lee S.H., Antov P., Kristak L., Reh R., Mardawati E., Santoso A. (2023). Latest Advancements in the Development of High-Performance Lignin- and Tannin-Based Non-Isocyanate Polyurethane Adhesive for Wood Composites. Polymers.

[77] Sari R.A.L., Lubis M.A.R., Sari R.K., Kristak L., Iswanto A.H., Mardawati E., Fatriasari W., Lee S.H., Reh R., Sedliacik J. (2023). Properties of Plywood Bonded with Formaldehyde-Free Adhesive Based on Poly(Vinyl Alcohol)–Tannin–Hexamine at Different Formulations and Cold-Pressing Times. J. Compos. Sci..

[78] Nordqvist P., Nordgren N., Khabbaz F., Malmström E. (2013). Plant Proteins as Wood Adhesives: Bonding Performance at the Macro- and Nanoscale. Ind. Crops Prod..

[79] Xiao G., Liang J., Wu Z., Lei H., Gong F., Gu W., Tu Y., Li D. (2023). A Composite Whole-Biomass Tannin–Sucrose–Soy Protein Wood Adhesive with High Performance. Forests.

[80] Lubis M.A.R., Park B.D., Hong M.K. (2020). Tuning of Adhesion and Disintegration of Oxidized Starch Adhesives for the Recycling of Medium Density Fiberboard. BioResources.

[81] Lubis M.A.R., Yadav S.M., Park B.D. (2021). Modification of Oxidized Starch Polymer with Nanoclay for Enhanced Adhesion and Free Formaldehyde Emission of Plywood. J. Polym. Environ..

[82] Tian W., Wang X., Ye Y., Wu W., Wang Y., Jiang S., Wang J., Han X. (2023). Recent Progress of Biomass in Conventional Wood Adhesives: A Review. Green Chem..

[83] Tabasum S., Zia K.M., Parveen B., Shahid M. (2022). Polyurethane Dispersions Prepared from Vegetable Oil and Their Application as Textile Finishes. Text. Res. J..

[84] Wang X., Nayanathara R.M.O., Leng W., Caldona E.B., Liu L., Advincula R.C., Zhang Z., Zhang X. (2022). Corrosion-Resistant Polyurethane Coatings from Structure-Homogenized Biorefinery Lignin through Fractionation and Oxypropylation. J. Agric. Food Res..

[85] Kachel M., Krawczuk A., Krajewska M., Parafiniuk S., Guz T., Rząd K., Matwijczuk A. (2023). Comparative Analysis of Vegetable and Mineral Oil-Based Antiadhesive/Hydrophobic Liquids and Their Impact on Wood Properties. Materials.

[86] Pang H., Ma C., Zhang S. (2022). Conversion of Soybean Oil Extraction Wastes into High-Performance Wood Adhesives Based on Mussel-Inspired Cation-π Interactions. Int. J. Biol. Macromol..

[87] Lee A., Deng Y. (2015). Green Polyurethane from Lignin and Soybean Oil through Non-Isocyanate Reactions. Eur. Polym. J..

[88] Abbas Z. (2020). Bio-Based Polyurethane Adhesives: A Review Bio-Based Polyurethane Adhesives: A Review. Dep. Appl. Chem. Gov. Coll. Univ. Faisalabad.

[89] Xiao X., Dong Y., Tang Z., Shi S., Gu L. (2023). Degradable, Anticorrosive, and Fluorescent Waterborne Polyurethanes from Vegetable Oil Internal Emulsifiers for Adhesives and Strain Sen Sor. Ind. Crops Prod..

[90] Gama N., Ferreira A., Barros-Timmons A. (2019). Cure and Performance of Castor Oil Polyurethane Adhesive. Int. J. Adhes. Adhes..

[91] Jasiūnas L., Peck G., Bridžiuvienė D., Miknius L. (2020). Mechanical, Thermal Properties and Stability of High Renewable Content Liquefied Residual Biomass Derived Bio-Polyurethane Wood Adhesives. Int. J. Adhes. Adhes..

[92] Lu Y., Zhang P., Fan M., Jiang P., Bao Y., Gao X., Xia J. (2019). Dual Bond Synergy Enhancement to Mechanical and Thermal Properties of Castor Oil-Based Waterborne Polyurethane Composites. Polymer.

[93] Kaur R., Singh P., Tanwar S., Varshney G., Yadav S. (2022). Assessment of Bio-Based Polyurethanes: Perspective on Applications and Bio-Degradation. Macromol.

[94] Purwanto E., Riadi L., Nathania Tamara I., Mellisha Ika K. (2014). The Optimization of Ozonolysis Reaction for Synthesis of Biopolyol from Used Palm Cooking Oil. ASEAN J. Chem. Eng..

[95] Bizet B., Grau É., Cramail H., Asua J.M. (2020). Water-Based Non-Isocyanate Polyurethane-Ureas (NIPUUs). Polym. Chem..

[96] Khatoon H., Iqbal S., Irfan M., Darda A., Rawat N.K. (2021). A Review on the Production, Properties and Applications of Non-Isocyanate Polyurethane: A Greener Perspective. Prog. Org. Coat..

[97] Heinrich L.A. (2019). Future Opportunities for Bio-Based Adhesives-Advantages beyond Renewability. Green Chem..

[98] Ang K.P., Lee C.S., Cheng S.F., Chuah C.H. (2014). Synthesis of Palm Oil-Based Polyester Polyol for Polyurethane Adhesive Production. J. Appl. Polym. Sci..

[99] Kaikade D.S., Sabnis A.S. (2023). Recent Advances in Polyurethane Coatings and Adhesives Derived from Vegetable Oil-Based Polyols. J. Polym. Environ..

[100] Barnwal B.K., Sharma M.P. (2005). Prospects of Biodiesel Production from Vegetable Oils in India. Renew. Sustain. Energy Rev..

[101] Prussi M., Chiaramonti D., Riccio G., Martelli F., Pari L. (2012). Straight Vegetable Oil Use in Micro-Gas Turbines: System Adaptation and Testing. Appl. Energy.

[102] Islam M.N., Rahman F., Das A.K., Hiziroglu S. (2022). An Overview of Different Types and Potential of Bio-Based Adhesives Used for Wood Products. Int. J. Adhes. Adhes..

[103] Marques A.C., Mocanu A., Tomić N.Z., Balos S., Stammen E., Lundevall A., Abrahami S.T., Günther R., de Kok J.M.M., de Freitas S.T. (2020). Review on Adhesives and Surface Treatments for Structural Applications: Recent Developments on Sustainability and Implementation for Metal and Composite Substrates. Materials.

[104] Zhang J., Wu Y., Zhang H., Yan T., Huang Y., Jiang J., Tang J.-J. (2021). Castor Oil-Glycerol-Based Waterborne Polyurethane Dispersions. Prog. Org. Coat..

[105] Eisen A., Bussa M., Röder H. (2020). A Review of Environmental Assessments of Biobased against Petrochemical Adhesives. J. Clean. Prod..

[106] Metzger J.O., Eissen M. (2004). Concepts on the Contribution of Chemistry to a Sustainable Development. Renewable Raw Materials. Comptes Rendus Chim..

[107] Cui S., Luo X., Li Y. (2017). Synthesis and Properties of Polyurethane Wood Adhesives Derived from Crude Glycerol-Based Polyols. Int. J. Adhes. Adhes..

[108] Hsissou R., Seghiri R., Benzekri Z., Hilali M., Rafik M., Elharfi A. (2021). Polymer Composite Materials: A Comprehensive Review. Compos. Struct..

[109] Alam M., Akram D., Sharmin E., Zafar F., Ahmad S. (2014). Vegetable Oil Based Eco-Friendly Coating Materials: A Review Article. Arab. J. Chem..

[110] Alcock T.D., Salt D.E., Wilson P., Ramsden S.J. (2022). More Sustainable Vegetable Oil: Balancing Productivity with Carbon Storage Opportunities. Sci. Total Environ..

[111] McDevitt J.E., Grigsby W.J. (2014). Life Cycle Assessment of Bio- and Petro-Chemical Adhesives Used in Fiberboard Production. J. Polym. Environ..

[112] Petrović Z.S., Hong D., Javni I., Erina N., Zhang F., Ilavský J. (2013). Phase Structure in Segmented Polyurethanes Having Fatty Acid-Based Soft Segments. Polymer.

[113] Wu Y., Qing Y., Wan H., Li X., Li X., Wang Y., Liu M., Yang S. (2023). Preparation and Characterization of Hybrid Resin from Used Urea-Formaldehyde and Isocyanate Resin. Int. J. Adhes. Adhes..

[114] Bartkowiak M., Czech Z., Mozelewska K., Kabatc J. (2020). Comparison between Thermal Crosslinkers Based on Melamine-Formaldehyde and Benzoguanamine Resin and Their Influence on Main Performance of Acrylic Pressure-Sensitive Adhesives as Tack, Peel Adhesion, Shear Strength and Pot-Life. Polym. Test..

[115] Marchione F., Chiappini G., Munafò P. (2022). Effect of Temperature and Relative Humidity on the Shear Performance of Double-Lap Adhesive Joints between Steel and Glass Adherends. J. Build. Eng..

[116] Viana G., Costa M., Banea M., da Silva L. (2017). A Review on the Temperature and Moisture Degradation of Adhesive Joints. Proc. Inst. Mech. Eng. Part L J. Mater. Des. Appl..

[117] Aristri M.A., Lubis M.A.R., Yadav S.M., Antov P., Papadopoulos A.N., Pizzi A., Fatriasari W., Ismayati M., Iswanto A.H. (2021). Recent Developments in Lignin- and Tannin-Based Non-Isocyanate Polyurethane Resins for Wood Adhesives—A Review. Appl. Sci..

[118] Norström E., Demircan D., Fogelström L., Khabbaz F., Malmström E. (2017). Green Binders for Wood Adhesives. Appl. Adhes. Bond. Sci. Technol..

[119] Mort R., Olson E., Thurber H., Jiang S., Vorst K., Curtzwiler G. (2022). Waterborne Polyurethane/Acrylic Adhesive Blends from Physaria Fendleri Oil for Food Packaging Applications. Sustainability.

[120] Nacas A.M., Antonino L.D., Chinellato A.C., dos Santos D.J. (2019). Nano Boron Nitride/Polyurethane Adhesives in Flexible Laminated Food Packaging: Peeling Resistance and Permeability Properties. Int. J. Adhes. Adhes..

[121] Bhakri S., Ghozali M., Cahyono E., Triwulandari E., Restu W.K., Solihat N.N., Iswanto A.H., Antov P., Savov V., Hua L.S. (2023). Development and Characterization of Eco-Friendly Non-Isocyanate Urethane Monomer from Jatropha Curcas Oil for Wood Composite Applications. J. Renew. Mater..

[122] Doley S., Dolui S.K. (2018). Solvent and Catalyst-Free Synthesis of Sunflower Oil Based Polyurethane through Non-Isocyanate Route and Its Coatings Properties. Eur. Polym. J..

[123] Pouladi J., Mirabedini S.M., Eivaz Mohammadloo H., Rad N.G. (2021). Synthesis of Novel Plant Oil-Based Isocyanate-Free Urethane Coatings and Study of Their Anti-Corrosion Properties. Eur. Polym. J..

[124] Dumont M.J., Kharraz E., Qi H. (2013). Production of Polyols and Mono-Ols from 10 North-American Vegetable Oils by Ozonolysis and Hydrogenation: A Characterization Study. Ind. Crops Prod..

[125] Omonov T.S., Kharraz E., Curtis J.M. (2011). Ozonolysis of Canola Oil: A Study of Product Yields and Ozonolysis Kinetics in Different Solvent Systems. JAOCS J. Am. Oil Chem. Soc..

[126] De Souza V.H.R., Silva S.A., Ramos L.P., Zawadzki S.F. (2012). Synthesis and Characterization of Polyols Derived from Corn Oil by Epoxidation and Ozonolysis. JAOCS J. Am. Oil Chem. Soc..

[127] Petrović Z.S., Wan X., Bilić O., Zlatanić A., Hong J., Javni I., Ionescu M., Milić J., Degruson D. (2013). Polyols and Polyurethanes from Crude Algal Oil. JAOCS J. Am. Oil Chem. Soc..

[128] Hazmi A.S.A., Aung M.M., Abdullah L.C., Salleh M.Z., Mahmood M.H. (2013). Producing Jatropha Oil-Based Polyol via Epoxidation and Ring Opening. Ind. Crops Prod..

[129] Saalah S., Abdullah L.C., Aung M.M., Salleh M.Z., Awang Biak D.R., Basri M., Jusoh E.R. (2015). Waterborne Polyurethane Dispersions Synthesized from Jatropha Oil. Ind. Crops Prod..

[130] Desroches M., Escouvois M., Auvergne R., Caillol S., Boutevin B. (2012). From Vegetable Oils to Polyurethanes: Synthetic Routes to Polyols and Main Industrial Products. Polym. Rev..

[131] Guo A., Demydov D., Zhang W., Petrovic Z.S. (2002). Polyols and Polyurethanes from Hydroformylation of Soybean Oil. J. Polym. Environ..

[132] Paraskar P.M., Prabhudesai M.S., Hatkar V.M., Kulkarni R.D. (2021). Vegetable Oil Based Polyurethane Coatings—A Sustainable Approach: A Review. Prog. Org. Coat..

[133] Agrawal A., Kaur R. (2019). Effect of Nano Filler on the Flammability of Bio-Based RPUF. Integr. Ferroelectr..

[134] Ionescu M., Radojčić D., Wan X., Petrović Z.S., Upshaw T.A. (2015). Functionalized Vegetable Oils as Precursors for Polymers by Thiol-Ene Reaction. Eur. Polym. J..

[135] Kong X., Liu G., Qi H., Curtis J.M. (2013). Preparation and Characterization of High-Solid Polyurethane Coating Systems Based on Vegetable Oil Derived Polyols. Prog. Org. Coat..

[136] Omrani I., Farhadian A., Babanejad N., Shendi H.K., Ahmadi A., Nabid M.R. (2016). Synthesis of Novel High Primary Hydroxyl Functionality Polyol from Sunflower Oil Using Thiol-Yne Reaction and Their Application in Polyurethane Coating. Eur. Polym. J..

[137] Ho L., Kim S.H., Kim B.K. (2008). Effects of the Hydroxyl Value of Polyol in Rigid Polyurethane Foams. Polym. Adv. Technol..

[138] Maisonneuve L., Chollet G., Grau E., Cramail H. (2016). Vegetable Oils: A Source of Polyols for Polyurethane Materials. OCL—Oilseeds Fats Crop. Lipids.

[139] Zain N.M., Ahmad S.H., Ali E.S. (2014). Durability of Green Polyurethane Adhesive Bonded Aluminum Alloy in Dry and Hydrothermal Ageing Conditions. J. Appl. Polym. Sci..

[140] Paz E., Narbón J.J., Abenojar J., Cledera M., del Real J.C. (2016). Influence of Acrylic Adhesive Viscosity and Surface Roughness on the Properties of Adhesive Joint. J. Adhes..

[141] Akram N., Gurney R.S., Zuber M., Ishaq M., Keddie J.L. (2013). Influence of Polyol Molecular Weight and Type on the Tack and Peel Properties of Waterborne Polyurethane Pressure-Sensitive Adhesives. Macromol. React. Eng..

[142] Nacas A.M., Ito N.M., Sousa R.R.D., Spinacé M.A., Dos Santos D.J. (2017). Effects of NCO:OH Ratio on the Mechanical Properties and Chemical Structure of Kraft Lignin–Based Polyurethane Adhesive. J. Adhes..

[143] Silva B.B.R., Santana R.M.C., Forte M.M.C. (2010). A Solventless Castor Oil-Based PU Adhesive for Wood and Foam Substrates. Int. J. Adhes. Adhes..

[144] Shi H., Magaye R., Castranova V., Zhao J. (2013). Titanium Dioxide Nanoparticles: A Review of Current Toxicological Data. Part. Fibre Toxicol..

[145] Kathalewar M.S., Joshi P.B., Sabnis A.S., Malshe V.C. (2013). Non-Isocyanate Polyurethanes: From Chemistry to Applications. RSC Adv..

[146] Singh M.K., Tewari R., Zafar S., Rangappa S.M., Siengchin S. (2023). A Comprehensive Review of Various Factors for Application Feasibility of Natural Fiber-Reinforced Polymer Composites. Results Mater..

[147] Unverferth M., Kreye O., Prohammer A., Meier M.A.R. (2013). Renewable Non-Isocyanate Based Thermoplastic Polyurethanes via Polycondensation of Dimethyl Carbamate Monomers with Diols. Macromol. Rapid Commun..

[148] Azra N.A., Atiqah A., Fadhlina H., Bakar M.A., Jalar A., Ilyas R.A., Naveen J., Sabaruddin F.A., Lim K.K., Asrofi M. (2023). Oil-Palm Based Nanocellulose Reinforced Thermoplastic Polyurethane for Plastic Encapsulation of Biomedical Sensor Devices: Water Absorption, Thickness Swelling and Density Properties. Appl. Sci. Eng. Prog..

[149] Broughton W. (2012). Testing the Mechanical, Thermal and Chemical Properties of Adhesives for Marine Environments. Adhesives in Marine Engineering.

